# Structuring and functionalization of non-metallic materials using direct laser interference patterning: a review

**DOI:** 10.1515/nanoph-2021-0591

**Published:** 2021-12-06

**Authors:** Lucinda Mulko, Marcos Soldera, Andrés Fabián Lasagni

**Affiliations:** Technische Universität Dresden, Institut für Fertigungstechnik, George-Baehr-Str. 3c, 01069, Dresden, Germany; PROBIEN-CONICET, Dto. de Electrotecnia, Universidad Nacional del Comahue, Buenos Aires 1400, Neuquén 8300, Argentina; Fraunhofer-Institut für Werkstoff- und Strahltechnik (IWS), Winterbergstraße 28, 01277, Dresden, Germany

**Keywords:** ceramics, composite materials, direct laser interference patterning, polymers, semiconductors, surface micro/nano-texturing

## Abstract

Direct laser interference patterning (DLIP) is a laser-based surface structuring method that stands out for its high throughput, flexibility and resolution for laboratory and industrial manufacturing. This top–down technique relies on the formation of an interference pattern by overlapping multiple laser beams onto the sample surface and thus producing a periodic texture by melting and/or ablating the material. Driven by the large industrial sectors, DLIP has been extensively used in the last decades to functionalize metallic surfaces, such as steel, aluminium, copper or nickel. Even so, DLIP processing of non-metallic materials has been gaining popularity in promising fields such as photonics, optoelectronics, nanotechnology and biomedicine. This review aims to comprehensively collect the main findings of DLIP structuring of polymers, ceramics, composites, semiconductors and other non-metals and outline their most relevant results. This contribution also presents the mechanisms by which laser radiation interacts with non-metallic materials in the DLIP process and summarizes the developed surface functions and their applications in different fields.

## 1 Introduction

### 1.1 Background

In the last decades, laser sources have become a valuable tool not only for research environments but also for highly-industrialized processes. This rapid deployment of laser technologies has been the consequence of the decrease in the costs of pulsed laser sources with output powers exceeding 100 W, paving the way for a plethora of advanced laser-based manufacturing processes such as cutting, welding, cladding, drilling, hardening, etc. [1], [2], [3], [4]. Moreover, laser-based surface micro/nanotexturing able to add or improve specific functionalities has caught the attention of the scientific community and the industry in order to develop value-added technical surfaces. For instance, producing deterministic microtextures using laser radiation has led to a wide variety of outstanding functions like superhydrophobicity [5], self-cleaning [6] and friction control [7] as well as to the development of microfluidic platforms [8] or microelectronic [9] devices. In the area of biomedical devices and tissue engineering [10], surfaces with antibacterial properties [11], biosensors/actuators [12] with therapeutic effects for dental prostheses [13] and neural scaffolds [14] have been developed too. Laser microprocessing technologies have also made their mark in the area of optoelectronics [15], energy storage, light scattering gratings [16] and others.

Among the laser surface structuring technologies [1, 17], direct laser interference patterning (DLIP) offers a remarkable combination of throughput, flexibility and resolution, making this technology suitable for surface structuring even for industrial manufacturing [18]. This technique has its origin in the pioneering works of Nebel and co-workers in the 1990s [19], [20], [21]. In their investigations, they overlapped multiple coherent beams to induce periodic arrays of crystallization seeds in amorphous silicon for obtaining a microcrystalline thin film for solar cells applications. Later in time, the DLIP method has been extensively used to pattern periodic textures on many materials, especially on metals like steel, aluminium, titanium alloys and nickel, among others, which are the most required by the large industrial sectors, such as automobile, aviation and energy. However, DLIP structuring of non-metallic materials, such as polymers, ceramics or composites, has been gaining attraction in emerging fields like optoelectronics, nanotechnology, biomedical devices and biomaterials [22]. It is therefore the motivation of this review to thoroughly gather the majority of the published works on this topic and sum up their most relevant findings. Moreover, the physico-chemical mechanisms by which laser radiation interacts with the non-metallic materials in the DLIP process are described along with a summary of the developed surface functions, which are linked to specific applications.

### 1.2 Fundamentals of direct laser interference patterning

The DLIP method relies on the superposition of at least two coherent beams on the sample, thereby producing an interference pattern. The overlapping *i*th beam can be considered as a polarized plane wave with an electrical field $\vec{E_{i}}$ oscillating in space and time given by:

(1) $$\vec{E_{i}}\left(\vec{r},t\right)=\vec{E_{0i}}\cos\left(\vec{k_{i}}\cdot \vec{r}-\omega t+\psi_{i}\right)\text{,}$$

where $\vec{E_{0i}}$ is the electrical field amplitude of the *i*th wave, $\left|\vec{k_{i}}\right|=2\pi /\lambda$ is its wave vector, *λ* is the wavelength, $\vec{r}$ is the spatial coordinates vector, *ω* is the angular frequency, *t* is time, and $\psi_{i}$ is the phase of the *i*th beam. Considering the principle of superposition of waves, the resulting electrical field upon overlapping *N* beams is:

(2) $$\vec{E}\left(\vec{r},t\right)=\overset{N}{\underset{i=1}{\sum }}\vec{E_{i}}\left(\vec{r},t\right)\text{.}$$

The intensity of the resulting interference profile can thus be expressed as [23]:

(3) $$I\left(\vec{r}\right)\propto <\sum_{i=1}^{N}\vec{E_{i}}{\left(\vec{r},t\right)}^{2}>\text{,}$$

where the angle brackets represent the time average over a time scale much larger than the period of the waves. If the wavelengths of the waves are the same, combining Eq. (2) with Eq. (3) gives the expression of the intensity profile of the interference pattern as [24]:

(4) $$I\left(\vec{r}\right)\propto \sum_{i=1}^{N}{\left|\vec{E_{0i}}\right|}^{2}+2\sum_{j<1}^{N}\sum_{i=1}^{N}\vec{E_{0i}}\cdot \vec{E_{0j}}\cos\left(\left(\vec{k_{i}}-\vec{k_{j}}\right)\cdot \vec{r}+\psi_{i}-\psi_{j}\right)\text{.}$$

**Figure 1:**
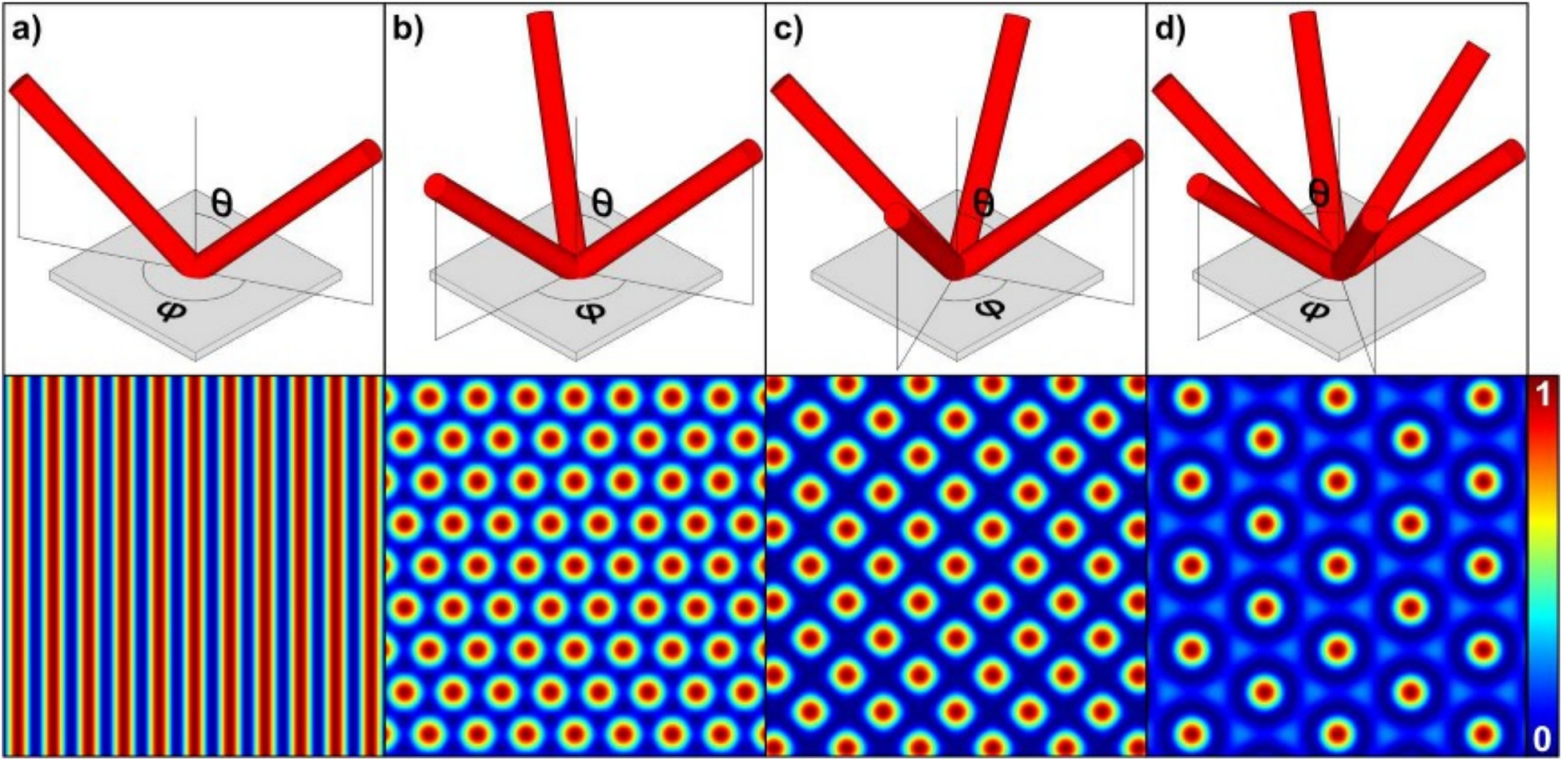
Schematic showing the set-up (upper row) and the resultant patterning (lower row) with (a) two, (b) three, (c) four and (d) six overlapping beams together with the calculated intensity of each interference pattern. Figures adapted from ref. [24] (© 2019, Springer International Publishing).

In this way, a periodical intensity distribution is formed with a spatial period (repetitive distance between the elements) given by the overlapping angle described by the wave vectors and wavelength. The shape of the pattern is defined by the number of overlapping beams, the phase shift between them and the polarization state. Figure 1 shows examples of different configurations of interfering beams and the resulting interference patterns. In these cases, the beams are arranged symmetrically with the same overlapping half-angle (*θ*) and azimuthal angles (*φ*), and all the beams have the same phase and polarization direction. The simplest interference pattern is achieved with two beams (Figure 1(a)) and has one-dimensional periodicity with a period defined by Λ=λ/(2sin(θ)) . In the examples with three (Figure 1(b)) and six (Figure 1(d)) beams, the intensity maxima give rise to periodic structures placed on a triangular lattice but with different spatial periods, namely Λ=2λ/(3 sin(θ)) and Λ=2λ/(√3  sin(θ)) , respectively. By overlapping four beams, a square array of periodic features can be obtained with a period Λ=λ/(3 sin(θ)) . Even more complex periodic interference patterns can be produced by the superposition of three or more beams with different phases and polarization directions [25].

The wavelength of the laser source must be selected according to the material spectral absorbance, so that the material can strongly absorb the laser radiation. It has to be mentioned though, that in some cases non-linear absorption mechanisms can be triggered by which the laser light can be absorbed in a material transparent to the laser wavelength [26]. At the maxima positions of the produced interference pattern the substrate can undergo local modifications, like recrystallization, oxidation, melting, ablation, swelling or even complex processes of reorganization [27], depending on the laser parameters and the optical and thermal properties of the material. To this end, the laser energy density, or fluence, must be higher than a certain threshold to activate the targeted physical modification, like melting, ablation, etc. Overlapping laser pulses at a given position increases the cumulated fluence on the material, which in turn induces a stronger modification of the material, e.g. wider or deeper ablated regions. Furthermore, the shape of the microtextures can be also modified by rotating the sample after a DLIP step a given angle and re-irradiating the surface. In this way, different periodicities and even multi-scale hierarchical textures can be fabricated. In summary, the synergy between the overlapping beams configuration, the structuring strategy and the materials properties can yield a large palette of achievable textures, shapes and thus, functionalities [28].

Several set-ups based on different optical components have been designed to generate the interference pattern on the samples. The advantages and limitations of each implementation approach have been described and discussed in detail elsewhere [25, 29], [30], [31], [32], [33], [34].

## 2 DLIP processing of polymers

The word polymer refers to a material formed by large molecules whose structure is composed of multiple repeating units. Polymers range from well-known synthetic plastics, such as polystyrene, to natural biopolymers, such as polyesters or polypeptides, and even macromolecules such as DNA can be considered polymers. Their broad spectrum of properties is associated with their molecular mass and molecular weight distribution as well as by their chain architecture, particularly by the amount of branching, resulting in unique physical properties such as high strength-to-weight ratio, high elasticity, viscoelasticity and tendency to form amorphous and semi-crystalline structures rather than crystals. In recent years, polymer materials science has advanced rapidly, giving rise to a wide variety of materials ranging from linear polymers with simple repeating units (e.g. polypropylene, polycarbonate, polyimide, among others) to more complex polymers with branched-chains (such as natural polymers as cellulose or three-dimensional macromolecules of arborescent construction or dendrimers) to composite materials (cross-linked polymers or hydrogels and block co-polymers, among others) [35], [36], [37].

Additionally, from a thermodynamic point of view, the glass transition temperature (*T*_g_) represents an essential indicator for the classification of a plastic compound as a thermoplastic or thermoset polymer. Thermoplastic polymers can be softened by heating (above *T*_g_) and hardened by cooling; thus they have the potential to be recycled and used multiple times. Typical examples of these linear polymers are polyethylene, polycarbonate and polyvinyl chloride. In contrast, thermoset polymers can only be molded once by heating; if they are heated again, they will not soften because the polymer chains have become intertwined. These polymers are cross-linked or highly branched molecules. Examples of this type are polyester resin, epoxy resin and silicones.

The growing demand for polymers has made it necessary to produce and process them with ever more accurate and faster methods. The unique properties of lasers have made them a preferable alternative to conventional machining for ultra-fine surface modifications [38]. This is especially attractive for fragile polymers that crack and rubber-like polymers that creep under the pressure of a conventional power tool [39]. Laser processing of polymers, such as cutting, trimming, marking, shaping and softening of polymers, has been done mainly with CO_2_, Nd:YAG and KrF excimer lasers [2, 17, 40, 41]. When it comes to micro and nanoprocessing of surfaces, DLIP arises as one of the best options in terms of operational versatility and structure uniformity. Due to the diversity of functionalization, coating, doping or composite formation possibilities offered by these materials, laser-polymer interactions are complex and multivariate.

This section provides an overview of current advances in the structuring of polymer surfaces by DLIP, from their laser interaction mechanism, through structuring on simple polymer surfaces to more complex advanced materials, describing in each instance their most relevant applications. One of the main advantages of thermoplastic polymers is related to their minimal chemical change during and after thermal processing, as well as their ductility and recycling potential, which is why they are preferred for use in laser processing as discussed in Sections 2.1 and partially in Sections 2.2.1 and 2.2.2. However, DLIP structuring has been reported on thermosetting polymers such as cross-linked polymers, polyurethanes and silicones as mentioned in Section 2.2.3.

### 2.1 Conventional polymers

The first polymeric materials structured by DLIP were thermoplastic synthetic polymers with linear (or quasi-linear) structure as films such as polycarbonate (PC), polyimide (PI), polyether ether ketone (PEEK), polyethylene terephthalate (PET), and several polyurethanes (PUs). These polymers are widely commercially available and are commonly used in many industrial applications, like packaging, pharmaceutical and food containers, transparent optical components or high-temperature mechanical components.

#### 2.1.1 Single-scale architectures

In 1987, Ilcisin and Fedosejevs [42] first described a technique for the direct production of holographic diffraction gratings on PET and PI films by irradiation with interfering KrF ns-laser beams, although with a slightly different configuration than the one used in current systems. In the 90s, Phillips et al. [43] published a manuscript in which periodic line structures with a period of 167 nm and line widths varying from 35 to 100 nm were produced on PI by direct ablation with a KrF laser using an interferometric configuration. In 1996, Karnakis and co-workers [44] carried out a series of studies on grating formation for optoelectronic applications using 248 nm and 193 nm excimer lasers, including an evaluation of laser-induced periodic surface structures and UV holographic techniques based on a phase mask to form lattices in polymers. Along these lines, in 1999 Lippert et al. [45] published a paper describing the formation of a nm-sized grating in polymers by laser ablation with an irradiation wavelength of 355 nm. A few years later, in 2003, Klein-Wiele and Simon [46] published an article in which they reported the fabrication of periodic nanostructures by interference of multiple beams and sub-picosecond laser pulses at 248 nm on polycarbonate surfaces. Later, in 2005 Mücklich et al. [47] reported for the first time the use of DLIP with Nd:YAG (266 nm) to control cell growth onto PET polymeric substrates using a two-beam interference setup, using the so-called beam splitter configuration (though at that time the technique was called laser interference lithography). This work sowed the seed for all subsequent development of DLIP in polymers.

During that period, the technique became widespread, particularly using non-polymeric substrates such as semiconductors, glasses, transparent oxides and metals [48], [49], [50], [51]. However, the mechanisms that enable absorption and, consequently, material ablation, in polymers are notoriously different. For example, absorption in metals is basically performed by the free electrons, and occurs in the first tens of nanometer from the surface [52]. On the other hand, in polymers absorption takes place due to the presence of chromophore groups consisting of *π*–π* conjugated double bonds or aromatic rings with delocalized *π* electrons [53, 54]. In 2007, Lasagni et al. [53] performed a comprehensive study to elucidate the patterning conditions by laser interference using a ns-laser in several biocompatible thermoplastic polymers of interest in the field of medicine. The study linked the chemical structure of each polymer, the mechanisms governing laser absorption and the working parameters of laser structuring such as wavelength (266 nm, 355 nm) and laser fluence. This is the initial in a series of papers that explore the versatility of the technique for structuring polymers films and their laser ablative mechanisms, which were supported by pioneering works [55], [56], [57]. Firstly, it is presented how the threshold laser fluence is directly related to the absorption coefficient of the polymer. For instance, polymethylmethacrylate (PMMA) fails to absorb laser radiation at both UV wavelengths used, which could be justified because of the chromophore group in PMMA is a C=O alkyl and the transition of the unpaired electron pair to the excited state of a *π*-bond (n–π*) is forbidden, therefore absorption is low. On the contrary, polymers containing aryl carbonyl groups such as PI or PC in which the *π*-bond is conjugated to the aromatic ring, can be structured by DLIP with UV radiation. The same can be explained by the concept of the cut-off wavelength of the studied polymers [24]. For example, PI shows an absorption cut-off at wavelengths above 350 nm, while the cut-off wavelength of PMMA is around 250 nm. Hence, since laser ablation only takes place when laser radiation is absorbed, a maximum absorbance at a defined wavelength is required.

**Figure 2:**
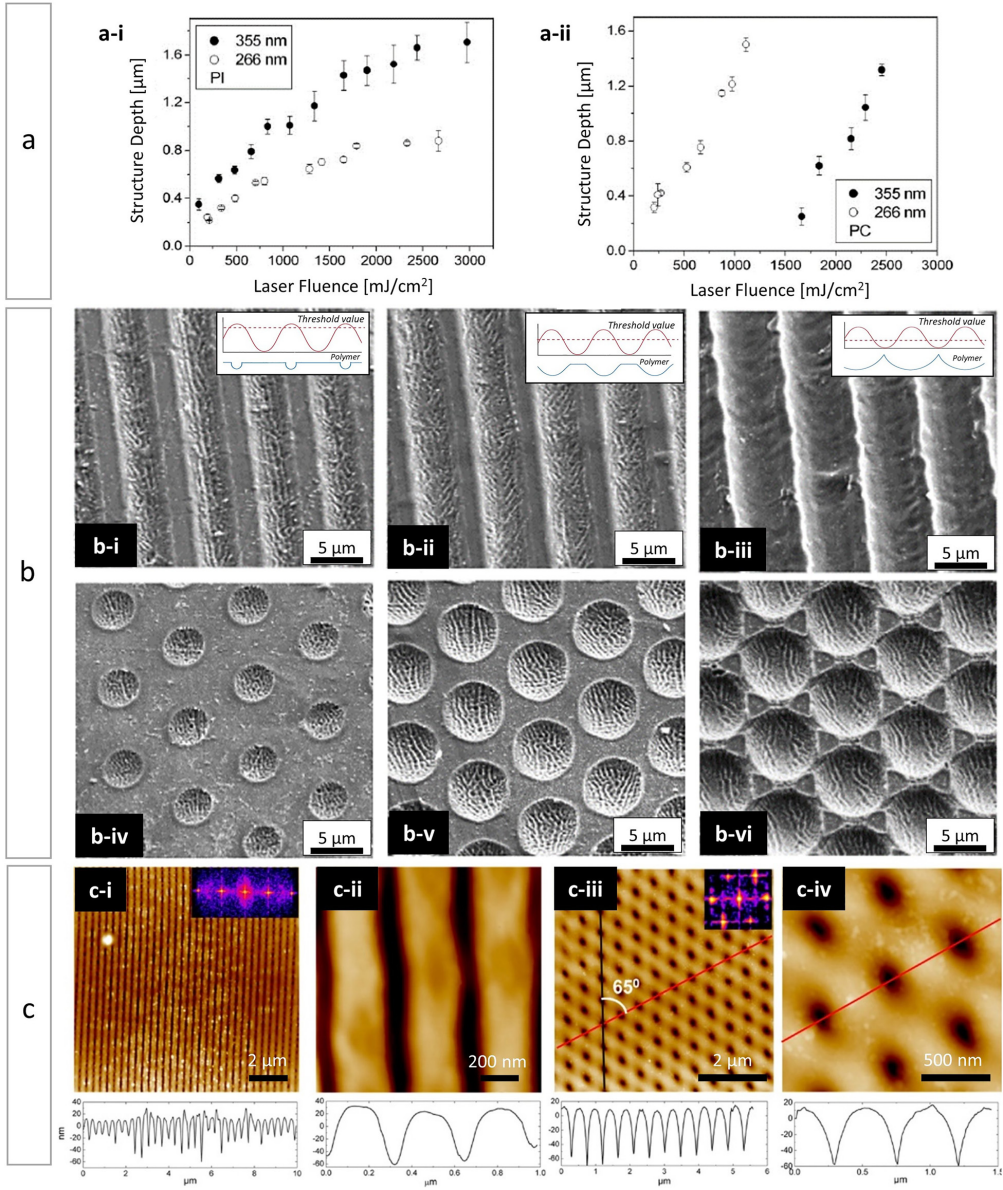
(a) Structure depth as function of the laser fluence for (a-i) PI and (a-ii) PC irradiated with laser radiation of 266 nm and 355 nm of wavelength, respectively. Line-type interference pattern. In all cases the period was 6.2 µm. (b) SEM images illustrating the evolution of the PI micropattern structured by DLIP at 355 nm from sinusoidal to U-shaped profile (scheme in between images where the dotted line is the laser fluence threshold value) for line-like at fluence (b-i) 312 mJ cm^−2^, (b-ii) 676 mJ cm^−2^ and (b-iii) 1023 mJ cm^−2^; and pillar-like at fluence (b-iv) 99 mJ cm^−2^, (b-v) pillar-like at fluence 277 mJ cm^−2^ and (b-vi) 854 mJ cm^−2^. (c) AFM images and corresponding profiles (bottom row) of PTT films structured by DLIP at 266 nm using two (c-i, ii) and three (c-iii, iv) interferences laser beams. Figures (a) to (b) adapted from ref. [53] (© 2007 WILEY‐VCH Verlag GmbH & Co. KGaA). Figure c adapted from ref. [68] (© 2014 American Chemical Society).

Besides, the researchers reported a correlation between laser fluence and the depth of structure [22, 58, 59]. This behaviour could be defined in two stages. After a certain threshold fluence value is reached (see Figure 2(a)), i.e. a minimum value below which there is generally no structure and which is closely related to the minimum energy required to achieve bond breaking [60], the depth of the structure increases linearly with the laser fluence. Moreover, if the photon energy is aimed at directly breaking the bond, chemical reactions are initiated, followed by decompression of the polymer and formation of gaseous products, which in turn can lead to the release of larger pieces weakly bound to the polymer and consequently to the almost complete removal of the upper layers of the substrates in the interference maxima positions [61]. In the second stage, the depth of the structure continues to increase with laser fluence, although at a lower rate. The existence of this last stage can be explained by the decrease of the effective absorption coefficient resulting from the shielding of microfragments of material released in the surrounding area of the surface or to the plasma created during the ablation process. This behaviour has been reported by other scientists not only for DLIP [24] but also for other related ablation processes in thermoplastics [52, 62], [63], [64]. For example, Jia et al. [63] reported an efficient strategy to perform locally controllable surface foaming on polypropylene interpenetrated with multilayer graphene through a pulsed near-infrared laser. Likewise, Rossa et al. [52] investigated surface modifications induced by irradiation with nanosecond laser pulses of ultraviolet and visible wavelengths on cross-linked hydrophilic polymeric materials. They found that microcrater-formation (strong material removal) was the dominant morphological change observed by ablation at 532 nm, while additional and less aggressive surface modifications (laser fluence: 0.9 J cm^−2^ and number of pulses (*N*): 5), mainly microfoams and roughness, developed in the ultraviolet at 266 nm.

Based on previous work linked to the dynamics of UV laser ablation in polymers [55, 57], Lasagni et al. [53] also discussed the mechanism that leads to UV-laser ablation in polymeric materials using ns-pulses. There are two broadly discussed mechanisms. On one hand, a photothermal mechanism, in which the ablation mechanism is associated with cumulative heating processes, as the energy of a single photon is insufficient to break a chemical bond, so the longer wavelength laser radiation is absorbed in the vibrational modes of the molecule. Once a bond absorbs enough photons, dissociation can be triggered. But while the energy accumulation takes place, the surroundings of the material absorb energy by vibrational energy transfer and are therefore susceptible to absorption and ablation [65]. On the other hand, a photochemical mechanism, by which the molecule dissociates after excitation to an unstable electronic state and ablation takes place after the material decomposes. This mechanism is distinguished by the formation of photofragmentation products such as atoms, small molecules and fragments of the polymer chain which are released from the surface at high speed, followed by a non-decompositional energetic relaxation back to the basal state of the material (intersystem crossing, quenching, etc.) [66]. These two mechanisms are extremes cases of a given process, as generally a combined effect drives the ablation of the material. The researchers relied on this principle to justify how the obtained structure depth depended on the laser fluence used [53]. Thus, in PI the structure depth was larger when a wavelength of 355 nm was used, while for PC the deepest structures were reached at 266 nm. In the last case, the absorption is due only to thermal processes since the *π*–π* transition of the aromatic ring is photochemically inactive, whereas at 355 nm the C=O chromophore is excited and the energy is relocalized to break the bond. Consequently, at 355 nm a photochemical contribution is added to the photothermal ablation process, which probably results in an increased ablation rate. Other publication [67] suggests that at shorter wavelengths the number of polymer fragments produced during ablation increases, the effective absorption coefficient is reduced and as a result, lower ablation rates are obtained. Figure 2(a) shows the two markedly different cases of UV structuring on two polymers. For PI (Figure 2(a-i)) there is no evident threshold fluence for either of the two working wavelengths. The linear slope, which linearly correlates fluence as a function of pattern depth, and a subsequent non-linear slope are observable. At 355 nm PI absorbs more than at 266 nm and hence the structure depth is larger at 355 nm for all applied fluences. Figure 2(a-ii), on the other hand, shows that PC requires a minimum fluence value below which patterning is not possible. This threshold value is particularly high for patterning at 266 nm approaching 1.5 J cm^−2^. A linear correlation between fluence and structure depth is then observed.

Figure 2(b) displays scanning electron microscope (SEM) images, extracted from another work by the same authors [53], of typical topographies obtained by two-beam interference (line-like) for three laser fluences (ordered in increasing order from left to right) for PI at 355 nm. The spatial period was 6.2 µm while the average width between ablated lines resulted in a range from 3.3 to 6.2 µm. They described the profile of the micropatterns as sinusoidal-trapezoidal at low laser fluences (300 mJ cm^−2^) where only a small area of the polymer reaches values above the threshold fluence (see respective schema below each Figure 2(b-i–iii)). As the laser fluence increases, more of the surface area exceeds the threshold fluence required to ablate the polymer, resulting in larger ablation regions, therefore at medium fluence values (600 mJ cm^−2^) the structure profile becomes sinusoidal (Figure 2(b-ii)), while for high laser fluences (1000 mJ cm^−2^) a U-shaped profile was observed (Figure 2(b-iii)). The authors reported comparable findings when the polymer samples were irradiated with a three-beam interference pattern (Figure 2(b-iv–vi)). In this case, the diameter of the ablated circular regions became progressively larger (3.6–7.2 µm) with increasing fluence (99–854 mJ cm^−2^). Furthermore, due to the geometry of the interference pattern at fluences of approximately 850 mJ cm^−2^, the circular patterns conformed into a periodic star-shaped pattern.

In another study, Martín-Fabiani et al. [68] reported the application of two- and three-beam DLIP configurations to trimethylene polythiophthalate (PTT) thin films. The experiments were performed with a single pulse of UV–laser radiation (266 nm) and fluences between 100 and 300 mJ cm^−2^. By changing the incidence angles, the fabrication of micrometer and submicrometer large-area polymer 1D grooves and cavities arranged in a hexagonal lattice was achieved. Figure 2(c) shows exemplarily some of the fabricated textures. Additionally, the mechanism of interference formation in polymer thin films was studied by inspecting different regions of the sample corresponding to different fluences due to the Gaussian-shape of the laser beam: from the edge of the irradiated region (minimum fluence) towards the center of the laser spot (maximum fluence). Thus, the researchers established three different regimes in the process. The morphology obtained outside the irradiated region corresponds to that expected for a PTT flat film. Inside the irradiated region, at the edge, slight signs of a periodical topography were observed, indicating that the process was initiated by linear absorption. Still at the edge, but closer to the center of the laser spot, characteristic interference patterning was observed. Finally, in the center of the laser spot, ablation holes appeared along with these patterns, indicating a non-linear response of the material, where above a certain laser fluence a complete removal of the material occurred.

Furthermore, another characteristic effect of laser ablation can be seen in the topographical DLIP profiles of Figure 2(c). When exposing a thermoplastic polymer to thermal annealing or UV radiation the polymer material flows from the unexposed regions (low surface energy) to the exposed regions (high surface energy) generating a three-dimensional topography due to the Marangoni effect, which describes the convective mass transfer due to surface energy gradients. In the case of DLIP in polymers, this phenomenon is more evident when using lasers of the order of nanoseconds and in the region close to the fluence threshold, in other words, in the boundary between the maximum and minimum of interference, generating a sinusoidal topography of varying thickness in this area.

Among the most widespread DLIP-related applications is the modification of the surface wettability of a material [69, 70]. For example, Estevam-Alves et al. [71] used DLIP to produce periodic surface structures on PU substrates with periods ranging from 0.5 to 5.0 µm using an ns-laser with 10 ns pulse duration. They investigated the influence of laser energy density on the quality and topographical characteristics of the produced micropatterns and how these influenced surface wettability. Two types of linear periodic patterns were produced on PU. Firstly, surfaces with spatial periods in the submicrometer-range (500–1000 nm) and structure depths of ca. 300 nm, and secondly PU-surfaces with spatial periods larger than 2.0 µm and depths between 0.88 and 1.25 µm. To characterize the wettability behaviour of patterned and untreated PU samples, the researchers performed water contact angle (WCA) measurements using the sessile droplet method. The results reported that the WCA of the slightly hydrophilic PU (81 ± 2°) increases significantly when it is patterned with a periodic structure of 3 µm and a depth/periodicity aspect ratio of 0.41, adopting a hydrophobic behaviour (102 ± 2°). This effect, already reported by others [72], can be explained by considering that the surface follows a behaviour according to the Cassie–Baxter model of heterogeneous wettability, in which air pockets are trapped between the microcavities of the solid surface and the liquid droplet. Hence, an originally hydrophilic material can be rendered liquid-repellent due to the microstructure of its surface. However, at a structured period of 500 nm and depths of about 200 nm, WCAs lower than those of the unstructured surface were obtained, which indicates that the surface is still hydrophilic and follows Wenzel’s theory. In this case, the original hydrophilic PU becomes more hydrophilic as the interfacial contact area increases (due to the topography) and homogeneous wettability occurs (the structure is not able to retain air at the solid–liquid interface).

Substrate topography has far-reaching implications for early bacterial adhesion and bacterial biofilm formation [73, 74] which is why the use of DLIP-modified surfaces has also been explored for the development of antimicrobial or bacterial aversive surfaces. The bacterial adhesion-topography interaction is intricate and non-trivial. On the one hand, it has been found that uncontrolled irregularities, such as voids or unshaped pores, facilitate bacterial adhesion and biofilm deposition, as it provides more favourable sites for colonization, whereas ultra-smooth polished surfaces do not favour bacterial adhesion and biofilm deposition [75, 76]. On the other hand, several scientists suggest that surfaces with rough topographies on the order of the nanoscale, particularly of the pillar or needle type, inhibit biofilm formation [77, 78].

**Figure 3:**
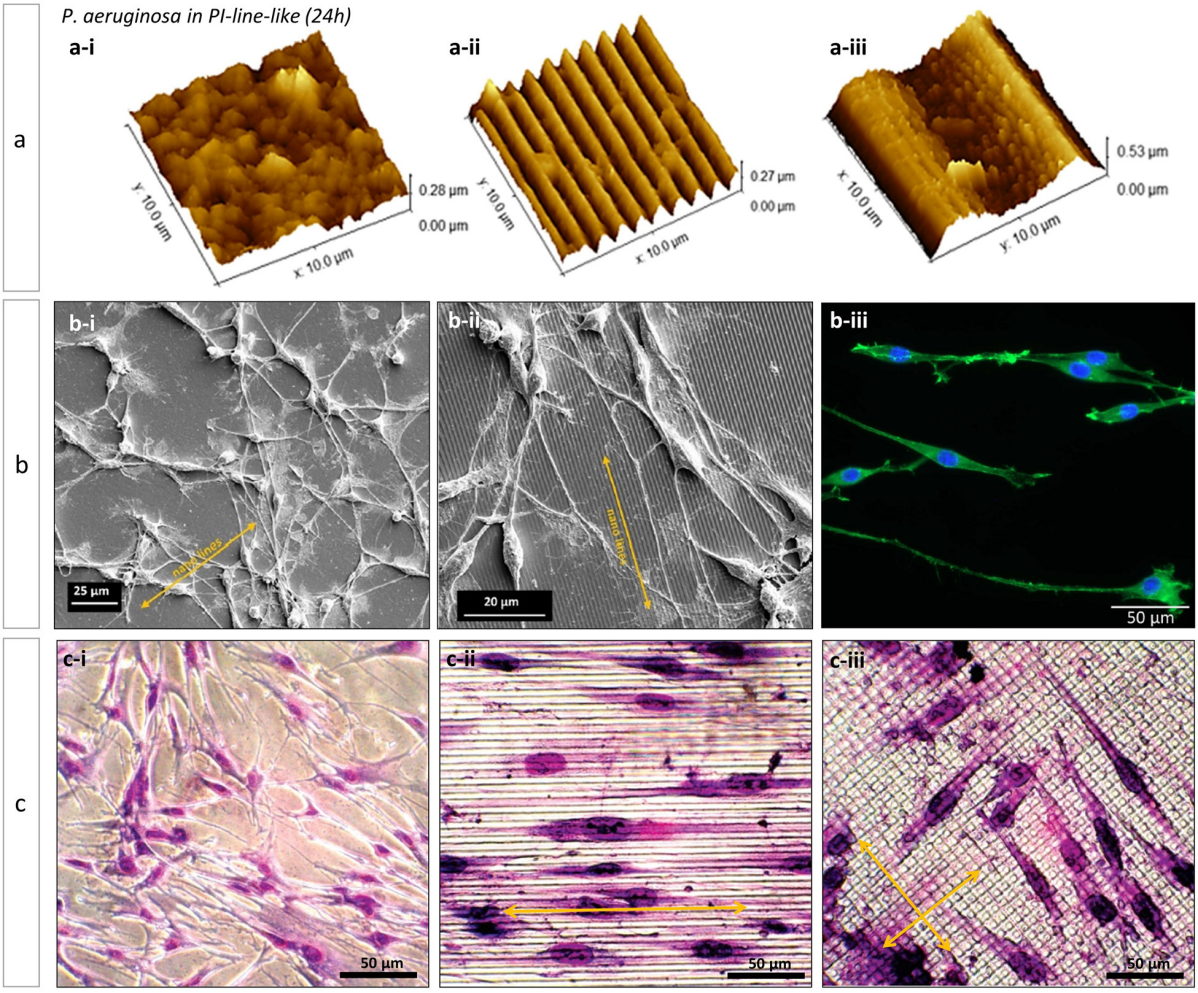
(a) AFM images of *P*. *aeruginosa* (24 h cultivated) in (a-i) PI unstructured, (a-ii) PI-line-like 1 µm and (a-iii) PI-line-like 10 µm films; (b) SEM images of B35 cells on DLIP-generated nanopatterns on PEEK with 1000 nm periodicity with (b-i) 50 nm deep grooves and (b-ii) 350 nm deep grooves. (b-iii) Fluorescence microscopy showing the alignment of B35 cells on PEEK-nanopatterns. Fluorescent staining: DAPI and phalloidin. (c) Optical images of HPF seeded on (c-i) flat PC films, (c-ii) PC films with a linear type structure with a period of 9 µm and (c-iii) PC films with a lattice-type structure with a period of 7 µm. In all cases the films were coated with collagen. Figures reproduced from: Figure 3a, ref. [72] (© 2020 Elsevier B.V.) Figure 3b, ref. [79] (© 2012 Laser Institute of America) and Figure 3c, ref. [81] (© 2005 American Chemical Society).

Werner and co-workers [74] reported a correlation of bacterial adhesion and biofilm formation (E. colli) with the dimensions of the structure on three photoresist resins (SU-8; APTES-(3-aminopropyl) triethoxysilane, (APTES) and TAF-amorphofluoropolymer, (TAF)). For the three tested cases, larger or equal periodicities to cell dimensions (>1 µm) seems to increase bacterial adhesion, while smaller periodicities (ca. 500 nm) decreased cell adhesion, despite contact time and hydrophobicity. In line with this, Cuello et al. [72] studied the effect of the size of linear-type DLIP-micro topographies on a PI film. The DLIP patterning was performed with periods of 1 μm, 2 µm and 10 μm. The antibacterial properties were evaluated by the effect on the growth of colonies of *Pseudomonas aeruginosa* bacteria. The results suggested that a periodic topography only imparts antifouling and biofilm reduction properties as long as the microstructure has periods ranging between 1 and 2 µm. For larger structures, no bacterial biofilm disruption was observed. Figure 3(a) shows atomic force microscopy (AFM) images of the PI-films contaminated with bacteria, it is notable how the bacillus bacteria appear to line up in the valleys of the structures when the size of the structure is slightly larger than the size of the bacteria.

Another application in the field of biomedicine of polymers structured by DLIP is the design of materials for cellular uptake and growth for tissue engineering. Neuronal cells, especially in the central nervous system (CNS), need guidance to reconnect after injury [14]. The strategy proposed by Bremus-Koebberling et al. [79] consisted of DLIP fabrication of neuronal scaffolds as a tool to guide cells in nerve reconnection. The work studied the geometrical features that influence cell behaviour, in particular the effect of depth and size of the structures in the nanometer range (spatial period: 100–1000 nm; depth: 100–600 nm), using two polymers films (PI and PEEK) as scaffold surfaces, which were subsequently embossing in PDMS in order to perform biological tests. Examination of cell orientation on the nanopatterns was carried out with B35 neuronal cells cultured in serum-free media for up to 96 h to expand the cell body extensions. The growth of these cells’ dendrites and axons on the linear nanogeometries was examined by SEM and fluorescence microscopy. Selected results are shown in Figure 3(b), in which a directional growth of the neuronal cells can be observed along with the pattern (Figure 3(b-ii)) while in other cases no such alignment occurs (Figure 3(b-i)). The researchers suggested that axonal alignment along the grooves occurs mainly when the so-called depth-to-width aspect ratio is 0.3 or greater. Analogous results were shown in another work but with a different cell line [80]. In this case, line-like structured surfaces (periods: 500 nm–10 µm) were fabricated on PI to produce topographical signals for cancer tumor cells. The cell-topography interaction was evaluated *in vitro* using mouse mammary adenocarcinoma cells. The results showed a cell growth guided by the direction of the patterning (more than 60% of the cell population is located in the grooves of the structure) for all tested structure sizes, with cells growing on patterns with a spatial period of 500 nm being the most aligned (up to 80%).

Similar results were obtained by Mücklich et al. [81] when studying the growth of human lung fibroblast (HPF) cells on DLIP-modified PC films. Figure 3(c) shows a series of bright field optical microscopies through which it is possible to compare cells grown on a flat (unmodified) and DLIP-structured PC surface in two different geometries. As shown in Figure 3(c-i), in the case of flat PC the cells do not show a specific growth direction, but are randomly oriented and often overlap with each other. In contrast, in the case of a surface with linear topography (Figure 3(c-ii)), the cells show a directional growth parallel to the line patterns. Finally, in the case of a pillar topography (Figure 3(c-iii)), the cells were again randomly oriented. Regarding cell shape, as opposed to cells seeded on the unmodified PC film which possesses a characteristic multipolar shape, HPFs seeded in linear and pillar-shaped patterns are mostly bipolar and spindle-shaped. Therefore, while the behaviour of flat and line-shaped PCs is very well defined, cells grown on pillar-shaped PCs seem to behave in an intermediate condition between the unmodified surface and the linear pattern, thus possessing a bipolar and spindle shape, but are randomly oriented on the surface.

The DLIP method is a scalable technology for treating large areas, which makes it interesting for the fabrication of organic photovoltaic (OPV) devices. It has been used to achieve structures such as gratings or pillars to improve the power conversion efficiency by lengthening the optical path of incident light within the absorber material, creating light-trapping geometries [82]. For example, Müller-Meskamp et al. [83] used DLIP to generate surface structures on flexible PET substrates with linear (4.7 μm period) and hexagonal (0.7 μm period) patterns that were subsequently coated with a poly(3,4-ethylene dioxythiophene) poly(styrene sulphonate) (PEDOT:PSS) and a ZnPc:C_60_-based small-molecule organic solar cell. All devices showed reasonable electrical performance, with an open-circuit voltage and fill factor comparable to those of glass or flat PET reference. It was further demonstrated that both the short-circuit current and power conversion efficiency were strongly improved by the surface structure and the higher light absorption in the active layer. Benchmarking the power conversion efficiencies against the reference cell in flat PET, a relative increase of about 5% was observed for the linear pattern, and a remarkable improvement of 21% for the hexagonal pattern.

**Figure 4:**
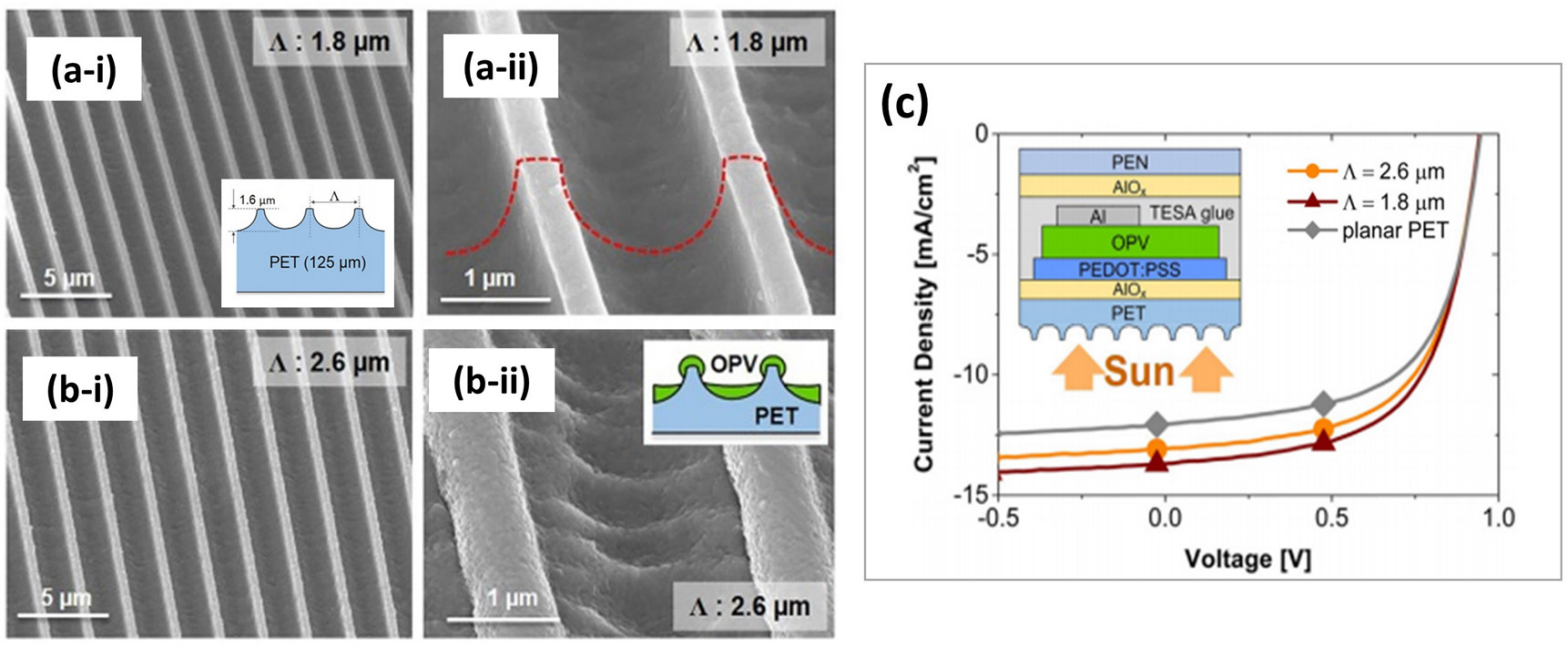
(a) and (b) SEM images of microstructured PET-DLIP (a-i) to (a-ii) with 1.8 µm period, (b-i) with 2.6 µm period wo/OPV multilayer, and (b-ii) with 2.6 µm period with OPV multilayer. In figures (a-i) y (b-i) the schematic patterning PET and PET-OPV is shown; (c) Current-potential curve for planar PET-OPV and DLIP PET-OPV at different periods, top left OPV multilayer scheme. Reproduced from ref. [82] (Licensed under a Creative Commons Attribution).

In a similar work, Leo et al. [82] designed a solar cell based on a small molecule blend of C_60_ and DCV5T-Me as an absorber layer deposited on structured PET. The micropatterns obtained are shown in Figure 4(a) and (b). The best performing device (line-like pattern with 1.8 µm period) achieved a power conversion efficiency of 7.7%, representing an improvement of over 16% relative to the reference. From the current–voltage curve in Figure 4(c), it can be seen that the short-circuit current density of the cells on structured PET increased strongly. Hence, the periodic topography seems to play a key role in the mechanism behind the increased efficiency by allowing for a greater light trapping effect in the solar cells, lengthening the absorption path and consequently concentrating more light on the absorber.

#### 2.1.2 Multi-scale and complex architectures

**Figure 5:**
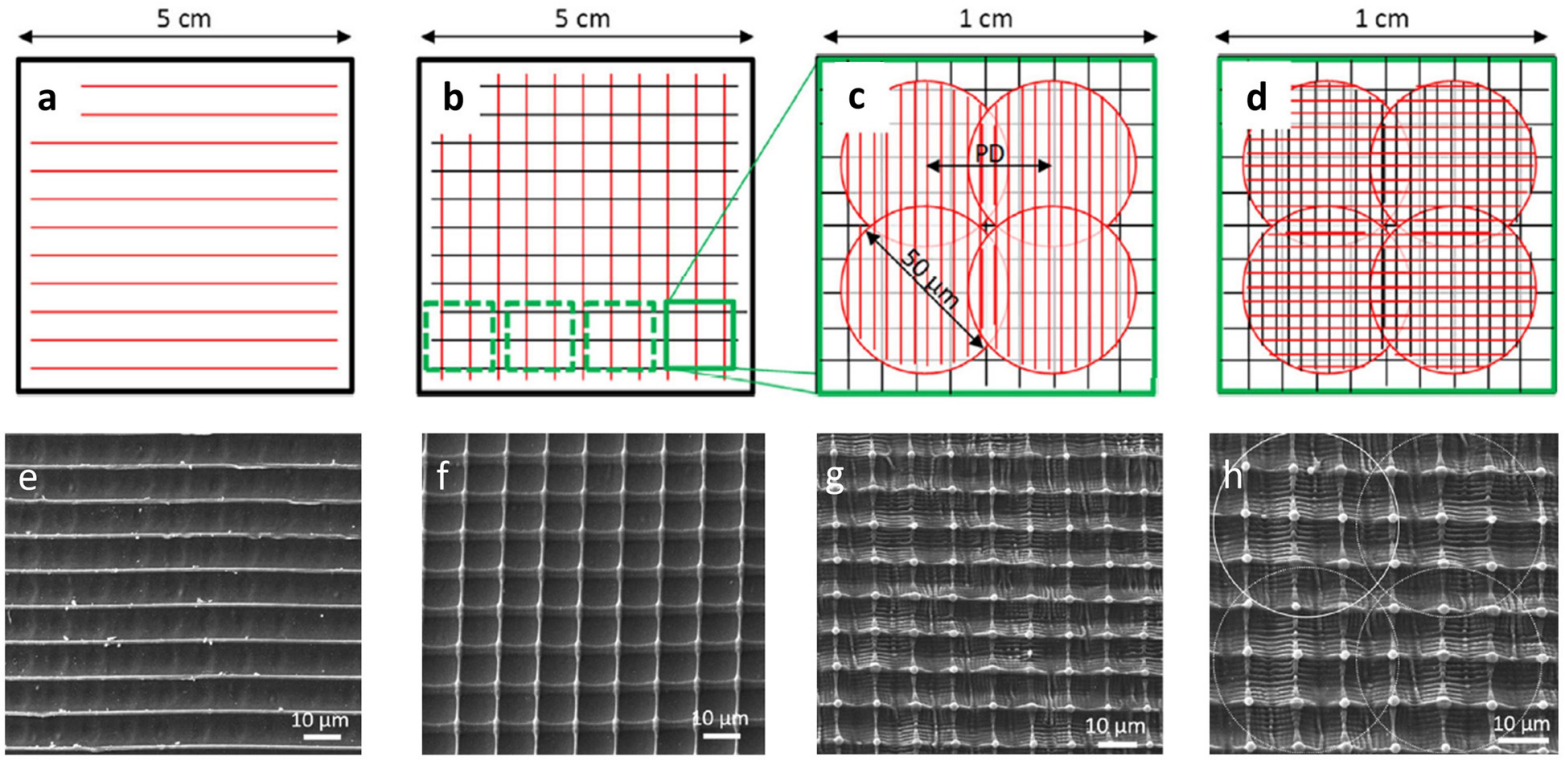
(a)–(d) Process steps for fabricating first the PET structures (the circles in white dotted lines in d indicate the size of each laser spot and the overlap between them. (e)–(h) SEM images of the structured PET each image corresponds to the process step indicated in each diagram immediately above in the top row. Figures reproduced from ref. [86] (© 2016 WILEY-VCH Verlag GmbH & Co. KGaA).

DLIP was explored to produce multi-scaled, or hierarchical, structures with two or three levels, mimicking the complex patterns present on numerous surfaces in nature [84, 85]. For example, Rößler et al. [86] presented the fabrication of three-level periodic structures on PET surfaces using DLIP with ns-laser to produce advanced diffractive optical elements. To achieve this, several sequential processing steps were used. Briefly, in a first step a linear periodic (10 µm spatial period, 266 nm, 10 Hz) distribution was generated and by a second step identical to the first one but rotating the sample 90° with respect to the initial position the high range pillar-like structure was achieved. Then, to obtain the short-range hierarchical structure, the process was repeated but with smaller structure periods (1–2 µm). The total diameter of each spot was 50 µm (referred in some publications as holographic pixel), whereby the distance between two spots was set to 35 µm. This last step gives the structure the third topographic sublevel. The sequence of steps by which the hierarchical structure is obtained is schematized in Figure 5(a)–(d), while the micrographs of the resulting topographies are shown in Figure 5(e)–(h).

**Figure 6:**
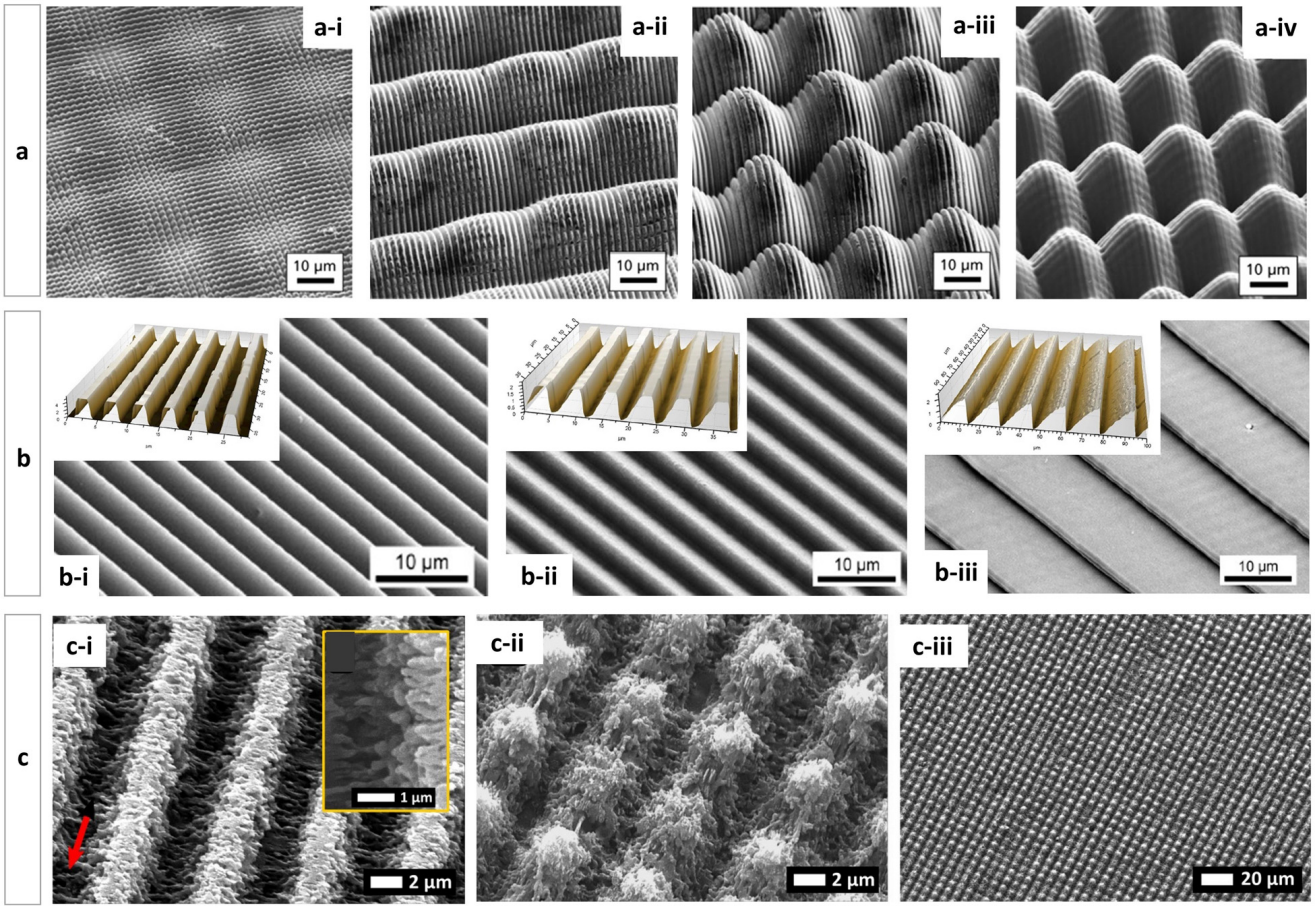
Three series of simple and hierarchical DLIP structures SEM images, portraying: Upper row (a): (a-i) PC-pillar-like (fluence: 0.51 J cm^−2^, *N*: 10 pulses), (a-ii) PC-lines-on-perpendicular-line-like (0.51 J cm^−2^, 90 pulses), (a-iii) PC-lines-on-pillar-like (1.63 J cm^−2^, 90 pulses) and (a-iv) PC-pillars-on pillars-like (1.63 J cm^−2^, 120) in all cases the structured period was 2 µm; middle row (b): (b-i) PI-inclined-lines-0° (b-ii) PI-inclined-lines-45° and (b-iii) PI-inclined-lines-75° (fluence: 0.51 J cm^−2^, *N*: 10 pulses) and confocal topography (upper left); lower row (c): (c-i) PI-line-like (fluence: 1.34 J cm^−2^, *N*: 10 pulses), (c-ii, iii) PI-crossline-like (fluence: 1.34 J cm^−2^, *N*_1st pulse_: 10, *N*_2nd pulse_: 5). The arrow in (c-i) indicates the polarization vector. These figures were adapted from ref. [87] (© 2018 Elsevier B.V.), [88, 95] (Licensed under a Creative Commons Attribution).

Similarly, Alamri et al. [87] presented several DLIP hierarchical structuring strategies on PC films employing a two-beam interference arrangement using a nanosecond ultraviolet laser (263 nm) and multi-pulse fabrication, aiming to fabricate hierarchical structures with selective wetting properties. As an example, the series of four SEM images in Figure 6(a) is shown. Figure 6(a-i) presents a hierarchical pillar-like structuring process (fluence: 0.51 J cm^−2^, *N*: 10 pulses for the second structuring) using the two-step fabrication strategy and the 90° between-step rotation of the sample presented above [87]. Figure 6(a-ii) shows a two-level structure with line-like DLIP structures on the lower level covered by orthogonal linear structures on the upper level (fluence: 0.51 J cm^−2^, overlap: 90 pulses, for the second structuring step) and Figure 6(a-iii) shows hierarchical structures with lines on the lower level, covered by pillars on the upper level (fluence: 1.63 J cm^−2^, overlap: 90 pulses, for the second structuring step). Finally, Figure 6(a-iv) shows a hierarchical pillar-on-pillar structure. For all structuring examples, the laser parameters for the first structuring process were kept constant (fluence: 1.48 J cm^−2^, structure period: 2.0 μm).

Later, Alamri et al. [88] presented an innovative technique to produce microstructures with complex non-symmetrical linear patterns based on the two-beam DLIP approach in order to fabricate surfaces with anisotropic functionalities. In this work, PI films mounted on an inclinable stage were irradiated with a 4 ns UV laser source (266 nm), producing linear structures with a period varying from 4.6 μm to 16.5 μm. The resulting saw-tooth topographies were obtained by tilting the sample with respect to the optical axis of the set-up. The topography tilt angles varied from 0° to 75°, thus achieving a well-defined and controllable inclination of the structure’s sidewalls. The study reports an exhaustive exploratory research on the evolution of the topography as a function of the inclination angles and the effect of the depth of the pattern as a function of the number of pulses. Examples of the structures produced at different angles (30°, 45° and 75°) at a laser fluence of 1.32 J cm^−2^ and 20 pulses are shown in the SEM micrographs of Figure 6(b) together with the corresponding optical confocal images. The SEM images indicate an inclination in the shape of the linear structures for angles of inclination greater than 30° and the presence of undercutting of the tilted walls. Furthermore, it was found that as the angle of inclination of the sample relative to the optical axis increases, the spatial period increases while the structure depth decreases.

In further cases, the hierarchical structures are not generated by sequential DLIP processing but by other simultaneous phenomena inherent to laser radiation, such as nanometric waviness known as laser-induced periodic surface structures (LIPSS) [89, 90]. Describing the mechanisms of ablation by LIPSS will be the scope of another review; here we will just briefly mention certain specific situations where LIPPS is somehow linked to the DLIP process in polymers. There are different opinions as to the cause of these wave-like structures but in general it responds to self-ordering processes of the material when it is exposed to ultra-short laser pulses. Some sources suggest that LIPSS originate from the interference of incident/refracted laser light with scattered or diffracted light near the surface [91, 92]. Others, that LIPSS occur due to shrinkage of the polymer layer during resolidification [93]. These structures are associated with irradiation with pulsed UV lasers with pulse lengths on the order of a few nanoseconds, fluences well below the ablation threshold and with a large number of laser pulses. The spatial period of the observed LIPSS is close to the laser wavelength. In the case of femtosecond laser, ripples are also observed on polymer surfaces at laser fluence above the ablation threshold, even with a low number of laser pulses [92, 94]. In this regard, Alamri et al. [95] presented the development of microstructured PI surfaces with multi-scale periodic patterns with two-dimensional symmetry by combining DLIP with LIPSS in a one-step process. A femtosecond laser source emitting at 1030 nm with a pulse duration of 500 fs and a repetition rate of 1 kHz was used for the experiments. As shown in Figure 6(c), grooves and pillars of several microns deep with very well-defined line-like structures could be obtained after 10 consecutive pulses on PI films. In general, the presence of LIPSS in polymers is associated with multiphoton absorption mechanisms [89, 91]. It was mentioned in that work that two-photon absorption process may be involved in the mechanism, as the material is transparent at the IR wavelength used [95]. The obtained patterns also showed some particular features. On one hand, the structure depth was significant deeper (ca. 3.9 µm) compared to patterns with the same period obtained with ns pulses and UV irradiation (up to 1.8 µm) [72] for which the material is a good absorber. On the other hand, at the positions of the interference maxima where a somewhat more disorganized bubble-like structure of the order of the nanoscale is observed. Besides, at the positions of the minima, some redeposition of the ablated material was reported.

**Figure 7:**
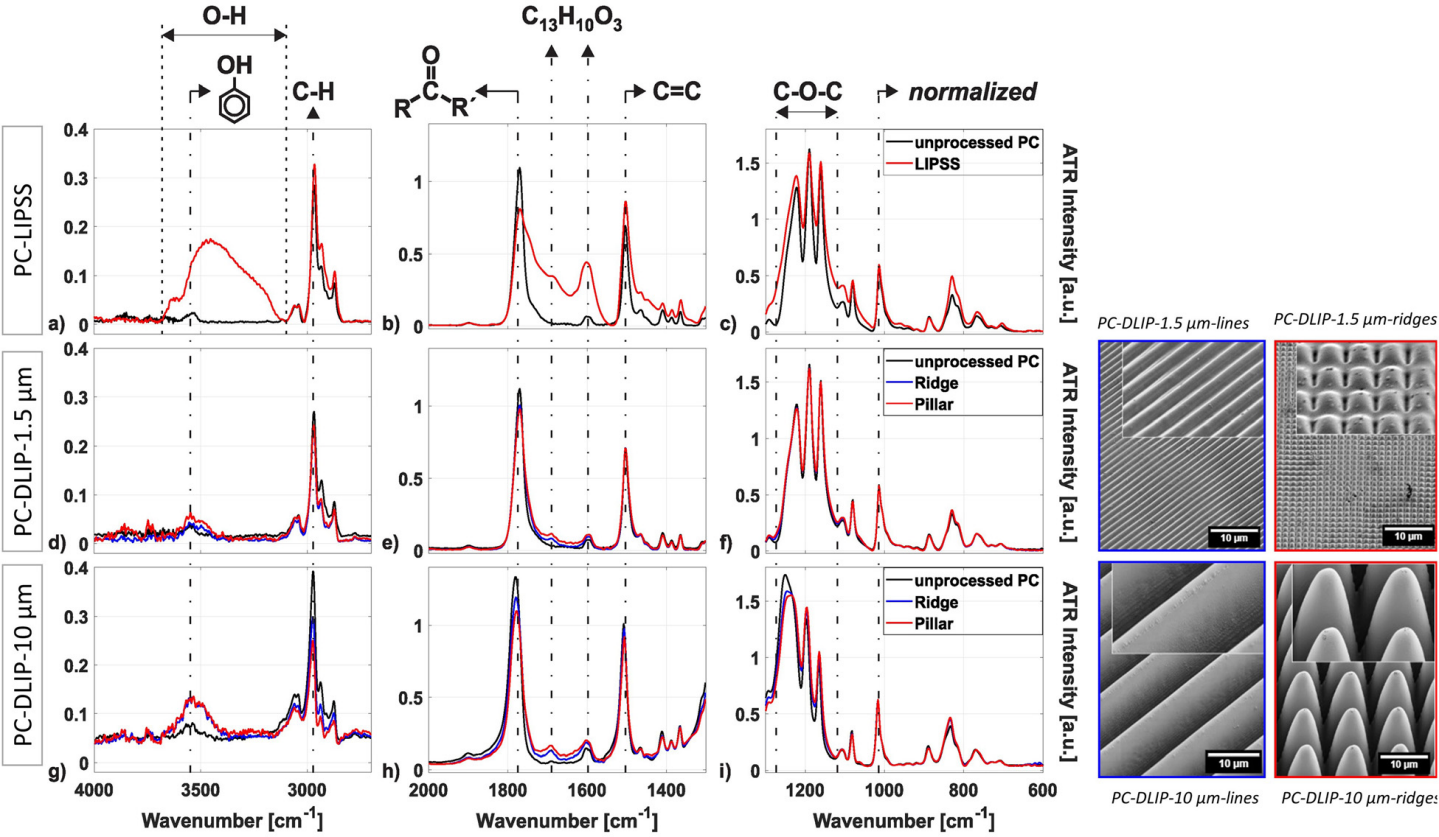
ATR-FTIR spectra of unprocessed PC (black curves) overlay with processed by (a)–(c) LIPSS (PC-LIPSS, red curves in and (d)–(f) PC-DLIP-1.5 µm ridge-like (blue curves) or pillar-like (red curves) and (g)–(i) PC-DLIP-1.5 µm ridge-like (blue curves) or pillar-like (red curves): In the left the corresponding SEM images. Figures reproduced from. Ref. [94] (Licensed under a Creative Commons Attribution).

Occasionally the LIPSS process does not occur simultaneously but can be incorporated in a second step after the DLIP process to produce controlled hierarchical structures. For instance, Mezera et al. [94] reported the generation of hierarchical micro/nano structures on PC surfaces by employing a two-step UV laser processing strategy. In a first step they used a DLIP nanosecond (3 ns) UV laser (266 nm) to achieve periodic structures on the order of the microscale and subsequently using a ps laser (7–10 ps, 350 nm) they patterned LIPSS with feature sizes of a few hundreds of nm. Afterward, by using FTIR-ATR, the chemical changes before and after laser irradiation were measured as well as the consequent degradation of the polymer. Figure 7 shows the spectra obtained for three different structuring conditions, the first row corresponding to LIPSS (PC-LIPPS), the second and third row to DLIP with a spatial period of 1.5 µm (PC-DLIP-1.5 µm) and 10.0 µm (PC-DLIP-10 µm), respectively. For a better understanding, SEM images of the measured structures are shown also in Figure 7. In all cases the spectra of the structured surfaces (red lines for line pattern or blue lines for pillar pattern) are compared with those of the unstructured surface (black lines).

In agreement with previous literature [72, 87], it was shown that in all cases, laser processing caused a partial degradation of the structure, particularly, in the C–H vibrational region (3000 cm^−1^), the carbonyl and C=C vibrational peaks (1790 and 1500 cm^−1^) and the C–O–C vibration (ca. 1120 cm^−1^ and 1280 cm^−1^). A relative increase in the band intensity was also observed, e.g. LIPSS processing gives rise to a broad absorption band related to the OH stretching region (ca. 3400 cm^−1^) and two increases, associated with the appearance of oligomers or short-chain polymers around 1630 cm^−1^ and 1690 cm^−1^ (see Figure 7(b)). Similarly, the processing of DLIP structures gives rise to absorption bands at 3550 cm^−1^ and 3500 cm^−1^ (see Figure 7(d)–(g)), which are attributed to free phenolic groups. On the one hand, comparing the relative intensity of the spectral bands of the LIPPS-structured and DLIP-structured PC, it is noticeable that the relative changes due to laser irradiation are more prominent for LIPSS than for any of the DLIP periods (e.g. when contrasting Figure 7(a)–(c) with Figure 7(d)–(f)). On the other hand, comparing DLIP structures with different periods, the spectra show a smaller band spread/shift due to the creation of DLIP structures with a narrow period (1.5 µm) compared to those with larger ones (10.0 µm). Moreover, it can be observed that pillar-like DLIP texturing affects more the degradation of PC than the fabrication of ridge-type DLIP structures. It was suggested that the different levels of polymer degradation can be attributed to the number of laser pulses irradiating a spot and the corresponding cumulative fluence, being higher for PC-LIPPS than for any of the PC-DLIP surfaces, and slightly higher for PC-DLIP-pillars than for PC-DLIP-ridges. Other similar study by the same group [87, 96] on hierarchical patterns on PC-DLIP presented similar conclusions.

**Figure 8:**
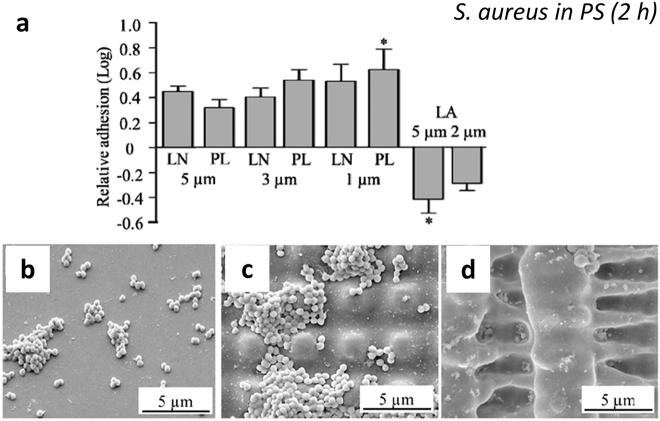
Qualitative assessment of *S*. *aureus* adhesion (static conditions, 2 h) to (a) reference PS-flat compared to (b) pillar-stamped PS (3 μm) and (c) lamellae PS; (d), relative adhesion (colony forming units (CFU) on structured and unstructured surfaces of *S*. *aureus* in PS with micrometric structures of lines (LN), pillars (PL) and lamellae (LA) at different periodic spacings. Figures reproduced from ref. [97] (© 2016 SPIE).

In addition to the applications mentioned above, multi-level structures have been used for the development of surfaces with antimicrobial/antifouling activity. For instance, Werner and co-workers [97] analyzed the adhesion behaviour of *Staphylococcus epidermidis* and *S*. *aureus* model bacteria on simple but also complex structures (e.g. lines, sheets, pillars) achieved by DLIP with periodic spacings from 0.5 μm to 5.0 μm fabricated on PI and polystyrene (PS) foils. These surfaces were characterized under *in vitro* and *in vivo* conditions. The results suggested that topographies have a significant impact on bacterial adhesion and they revealed different behaviours according to the geometry of the 3D-structures. Thus, after 2 h of bacteria culture (see Figure 8(a)), line (LN) and pillar (PL) patterns enhanced *S*. *aureus* adhesion in PS films, while complex microtopography in the form of lamellae reduced *S*. *aureus* attachment under static and continuous flow culture conditions. Curiously, the textured lamellar (LA) surfaces retained the ability to inhibit *S*. *aureus* adhesion when the surface was coated with human serum proteins and maintained their non-adhesive properties when the material was implanted subcutaneously in an *in vivo* model. As an example, SEM images of PS surfaces contaminated with *S*. *aureus* are shown in Figure 8(b)–(d). While large bacterial clusters spread over the entire surface of the structured PC pillars (Figure 8(c)), small bacterial clusters dispersed on complex laminar-like patterns (Figure 8(d)) as well as on the flat PC (Figure 8(b)). These studies in connection with the studies mentioned in Section 2.2.1, suggest a dependence of the adhesion and orientation of the bacterial cells on the periodicity and geometry of the DLIP-surfaces. Thus, it was possible to identify two well-defined cases: (i) for structures with dimensions larger than the cell size, the cell-substrate contact area increases, which contributes to high bacterial adhesion (similar to that of the smooth surface), (ii) for topographies with structure periods similar or smaller than the bacterial cell size, and particularly when the surface geometry is pillar-like, the contact area between the cell and the substrate was restricted, which tends to lead to lower cell adhesion. However, much remains to be elucidated and other factors must be considered, including contact time, the type and shape of the microorganism and the surface chemistry, among others. For example, in the case of spherical cells (*Staphylococcus* bacteria), cell retention was lower, while rod-shaped cells (*Pseudomona *and *Escherichia* bacteria) tended to settle in the cavities of the structure, making biofilm formation more difficult. Regarding surface chemistry, in Section 2.2 will be discussed the combined effect of surface structure an antibacterial coating in composite materials.

Subsequently, other works reported more sophisticated combinations of structures on these materials. For example, Rößler et al. [98] proposed a bilayer system consisting of a PMMA and PC film that was irradiated with a nanosecond pulsed laser at a wavelength of 263 nm. The top layer of PMMA is transparent to the used laser wavelength and thus the irradiated light was absorbed in the bottom layer of PC layer, which produced a periodic structure entrapped between the two layers. To study the in-volume structure, the authors used a cross-section polisher operated with argon ions combined with SEM/EDX analysis. Periodic round cavities, produced by the decomposition of PC, with a feature size of 500 nm were observed at the interface between the polymers. This in-volume patterning approach has been proposed for the fabrication of long-lasting holographic security devices, e.g. in credit or personal cards.

### 2.2 Composite and advanced polymers

A composite is defined as a material that involves the interaction of at least two constituent materials, yielding new or enhanced properties. An example of advanced composite polymer are materials composed of a transparent structural polymer and a chromophore polymer or agent with poor mechanical properties (i.e. a thermolabile polymer or a stable polymer as a colloidal dispersion) that is deposited, adsorbed, grafted, etc. onto the former (PolCrom@PolSupp). Conductive polymers as well as cross-linked polymers and block co-polymers will be considered as advanced materials in this review due to their extraordinary properties and their potential for novel applications, as will be discussed below.

#### 2.2.1 Chromophore-embedded polymers

A major concern about laser structuring in polymers is linked to their inherently low absorptivity in the VIS-IR wavelength range, which makes it intricate to structure by ns-lasers at wavelengths longer than 260–266 nm. This, in turn, struggles with the low pulse stability of UV lasers compared to longer wavelength lasers, especially at the scale-up stage of the process [99]. To overcome this drawback, several authors have documented the doping of transparent polymers with chromophore agents or photosensitizers such as azo dyes [100], conductive polymers [101] or fluorescent nanoclusters [102]. The dopants reduce the ablation threshold and increase the quality of ablated features.

**Figure 9:**
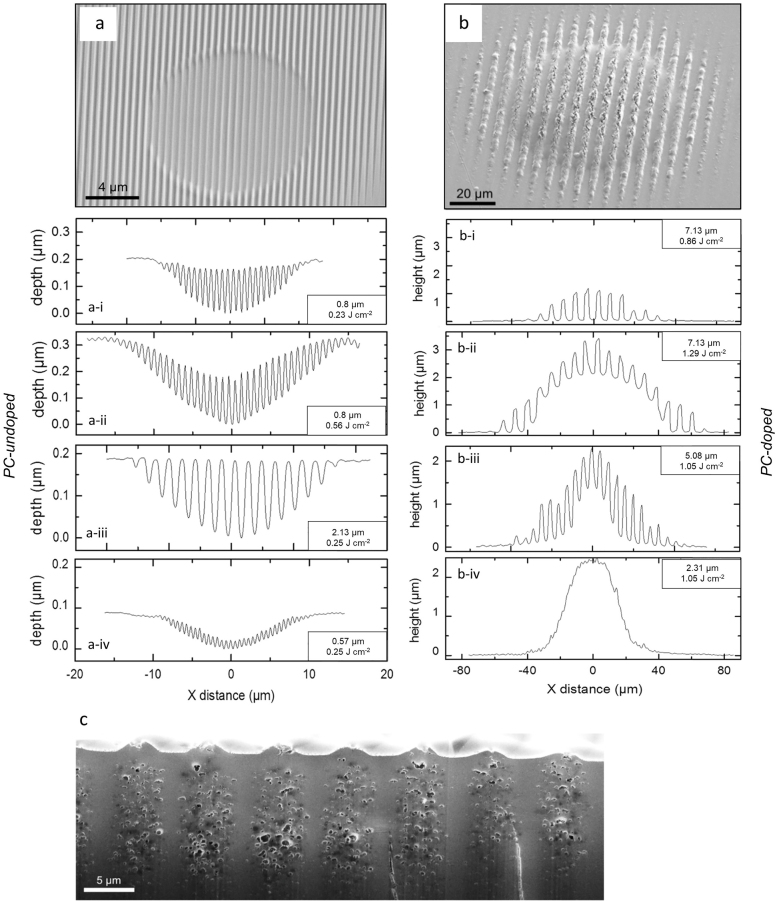
Left column: PC-undoped-DLIP line-like at a wavelength of 263 nm (a) SEM image of one Gaussian spot (pulse duration 3 ns, fluence 3.5 J cm^−2^, spatial period 0.56 µm), the shaded region forming a circle corresponds to the center of the laser spot where there is a depressed zone (zone of higher fluence) while the surrounding zone corresponds to the area of lower fluence. Confocal microscope profiles at a fixed period but varying the laser fluence (a-i, ii) and fixed fluence but varying the spatial period (a-iii, iv). Right column: PC-doped-DLIP line-like with swelling at 1053 nm (b) SEM image of one Gaussian spot. Confocal microscope profiles of at a fixed period but varying the laser fluence (b-i, ii) and fixed fluence but varying the spatial period. (a-iii, iv). The black box in each graphic indicates the fluence and the spatial period of structuring. (c) SEM-FIB image of a swelled PC-doped-DLIP line-like (1053 nm, fluence 1.3 J cm^−2^, period 7.3 µm). Reproduced from ref. [22] (© 2017, Optical Society of America).

To analyze this behaviour, Alamri and Lasagni [22] described a series of four measurements to develop an empirical model comparing transparent PC (PC-undoped) and pigmented (PC-doped) PC substrates treated by ns-DLIP at two markedly different wavelengths, namely UV (263 nm) and IR (1053 nm). Depending on the used laser processing conditions, the type of material as well as the spatial period of the interference pattern, four different behaviours were identified. The interference phenomenon combined with ablation can be well exemplified by PC-undoped foils irradiated with UV laser (Figure 9(a)). The results revealed that well-defined line-like patterns, which perfectly fitted the periodic intensity distribution, were obtained only for a small range of laser fluences (<0.1 J cm^−2^). For higher laser fluences, the interference phenomenon is added to the laser ablation so that the structure fades, giving rise to a partially structured volume. The same effect was observed keeping the laser fluence constant at a mean value (0.25 J cm^−2^) and exploring systematically different structuring periods, for periods below 1 µm (see Figure 9(a-iv)). The Gaussian shape of the ablation is to be expected due to the Gaussian intensity profile of the laser radiation (see Figure 9(a-i–iv)) [103]. Experiments of PC-undoped irradiated with IR reported no interaction with the laser over a wide range of fluence.

PC-doped structured at 1053 nm (Figure 9(b)) corresponds to the less frequently swelling phenomenon, also reported by some authors as foaming [52], combined with interference. When the polymer interacts with laser light some polymer chains can be excited and dissociate into gaseous by-products inside the material producing pores that do not reach the surface of the material and thus increasing the local volume [104]. This represents a clear example of a thermal ablation mechanism as mentioned in Section 2.1.1. The dynamics of pore formation can be considered independent of the interference period, for a fixed laser fluence and wavelength. Therefore, when the dimension of the expanding pores is smaller than the interference spatial period, the pores interconnect with each other forming non-periodic structures. This behaviour can be observed in the micrograph of Figure 9(b) and in the profile plots presented in Figure 9(b-ii and iii). As in the case of ablation, there is a window of fluence and structure periods where this particular phenomenon is observable. For low laser fluence and especially for large spatial periods, the polymer only swelled at the interference maxima, while for shorter structure periods or higher fluence the swelling effect predominated and no clear interference fringes were observed (see Figure 9(b-iv)) [22].

Next, PC-doped substrates were irradiated with UV laser radiation. Hither, the authors reported a swelling mechanism at fluences slightly above the threshold fluence, although not as evident as in the experiments with a laser wavelength of 1053 nm. At higher fluences the substrate was locally ablated at the positions of the interference maxima, obtaining results similar to those of the transparent material treated with UV radiation. At this wavelength, both the matrix and the dye can absorb the laser radiation and, therefore, dual ablation-expansion behaviour combined with the interference process is to be expected. Furthermore, in order to study the swelling phenomenon in depth, an SEM images of focused ion beam (SEM-FIB) milled cross-cuts of doped polycarbonate modified by DLIP (laser fluence 1.3 J cm^−2^, spatial period 7.13 μm) were presented. The image (Figure 9(c)) showed that micropores form specifically at laser interference maxima. They suggested that the dynamics of pore creation and expansion is independent of the interference period, for a fixed laser fluence and wavelength. Therefore, when the size of the expanding pores is smaller than the spatial period of interference, the pores quench, creating non-periodic structures.

Another example is the work reported by Broglia et al. [105] on which polystyrene doped with azo-colorant by DLIP at 355 nm was structured. The colorant incorporation pursuits not only to structure PS but also to achieve a more uniform and controlled structure compared with pure polystyrene. An azo-dye was selected because of its photosensitive properties and its ability to absorb light with high absorption coefficients in the spectral range between 300 and 400 nm, given by the presence of the azo group (–N=N–) in its structure. The range of working fluences required to produce sub/micro-arrays was between 0.2 and 1.0 J cm^−2^. The results of these studies indicated that to obtain linear-like textures in the sub-micron range (ca. 500 nm) a threshold fluence of 0.3 J cm^−2^ was needed. However, under these irradiation conditions the system must also handle the swelling phenomenon which produces localized swelling of the material and inhibits the development of regular periodic sub-micron arrays. Also, they studied the formation of different microstructures by DLIP on PS films with azo compound 2-anisidine (PdS) as a function of fluence. For this, a classical two-beam configuration was used to obtain linear interference patterns. A Nd-YAG ns-laser was used with a wavelength of 355 nm (triple harmonics). Laser ablation can then take place by either or both of these two phenomena: (i) the azo group absorbs the light and decomposes releasing gaseous nitrogen, which subsequently helps to remove the solid material from the ablated region; (ii) the heat absorbed by the dye is transferred to the polymer causing sublimation and/or rupture of the polystyrene chain. The first phenomenon is associated with the aforementioned photochemical absorption mechanism, while the second one depends on the thermal conductivity of the polymer and its ability to absorb heat from the dye. The systematic study at different fluences also showed the presence of pores in the zones of maximum interference that disappeared when structuring the surface at higher fluences.

**Figure 10:**
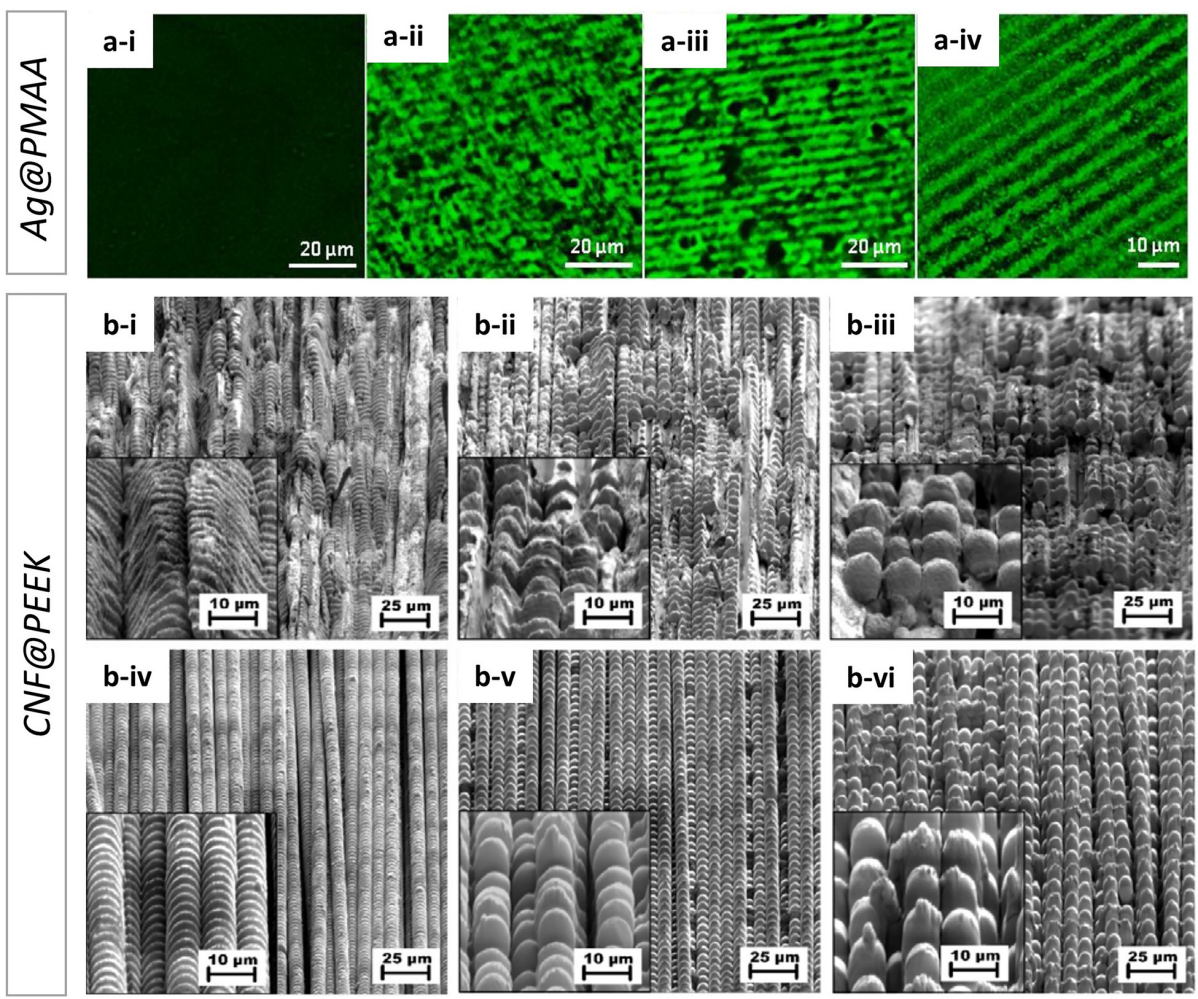
Ag@PMAA: Confocal microscope images Ag@PMAA before (a-i) and after processing by DLIP with increasing number of pulses *N*: (a-ii) *N* = 1 (a-iii) *N* = 3 and (a-iv) *N* = 5; CNF@PEEK: SEM analysis of surface structures fabricated with IR at laser fluence of 2.86 J cm^−2^ (first row) and 2 J cm^−2^ UV (second row) radiation with increasing *N*, spatial period Λ and with inserts of higher magnification images: (b-i) Λ = 2 μm, *N* = 200, (b-ii) Λ = 4.3 μm, *N* = 200, (b-iii) Λ = 8.5 μm, *N* = 200, (b-iv) Λ = 2 μm, *N* = 200, (b-v) Λ = 4.3 μm, *N* = 200, (b-vi) Λ = 8.5 μm, *N* = 200. Figures reproduced from ref. [106] (© 2019 Elsevier B.V.) and ref. [107] (© 2019 Elsevier B.V.).

In a similar study, active chromophore agent as nanoparticles or clusters embedded in a polymeric matrix and arranged in the form of DLIP structured films had been reported. Mulko et al. [106] described the use of DLIP at 355 nm with a ns-laser to pattern PMAA doped with photoreductive fluorescent silver nanoclusters surfaces (Ag@PMAA) as well as to induce nanocluster formation and subsequent laser patterning on unreduced silver polymeric films (Ag+@PMAA). The latter system presented the most relevant results as a single laser pulse allowed to create not only the line-like pattern on the surfaces but simultaneously generated the photoreduction of silver. As is observed in Figure 10(a-ii–iv), Ag+@PMAA once structured by DLIP (and simultaneously photoreduced) showed a highly ordered pattern of fluorescent lines after treatment with 1, 3 and 5 laser pulses that ablated the material, obtaining successively broader patterns. For sake of comparison, Figure 10(a-i) shows that in the case of the pristine material Ag+@PMAA film (non-irradiated) did not produce fluorescence in the detected spectral range, demonstrating that the nanoclusters were produced as a result of the interaction of the Ag+@PMAA film with the laser pulse.

Hauschwitz et al. [107] addressed the production of functional hierarchical microstructures by DLIP in carbon fiber (diameter of 5 μm) reinforced polymer composites (CNF@PEEK) in order to produce superhydrophobic surfaces. Taking advantage of the strong absorption of the carbon fibers in the near-infrared and of the supporting polymer in the UV, two-beam DLIP using an ultraviolet (263 nm) or infrared (1053 nm) ns-laser source was employed to produce well-defined and melt-free hierarchical microstructures in carbon fiber reinforced plastics. Since the center of the DLIP pixel has a higher fluence due to the laser intensity Gaussian distribution, dual-scale hierarchical structures in the fibers with large pillars (60 µm), generated by the DLIP pixel size, combined with micropillars, generated by the interference lines and were produced in a one-step process. The interesting finding of this work underlies the different structures that can be clearly observed by changing the working wavelength and obtaining a plethora of structuring possibilities. Meanwhile, the structuring of CNF@PEEK by IR-DLIP in the ns regime was mainly considered as a photothermal process resulting in an abrupt heating of the carbon fibers that was rapidly transferred to the surrounding area, leading to melting and ultimately vaporization of the polymer matrix around the heated fibers. Differently to IR-DLIP processing, no cracks or detrimental thermal effects were observed during UV-DLIP processing in the available laser power range. Due to the high absorption of the polymer at the wavelength used, fewer pulses per unit area were required for structuring. By increasing the number of pulses per spot, the polymeric matrix vaporizes due to the heat input from the fibers in a similar way as described for the IR treatment. However, the formation of hierarchical structures was observed only for specific cases (period 1.5 μm and pulse overlap larger than 99%). The SEM images in Figure 10(b) show the structures obtained for both IR-DLIP (i–iii) and UV-DLIP (iv–vi). In the former, the presence of cracked and damaged areas is evident, as well as the non-trivial formation of structural shapes such as ladder-like structures. For CNF@PEEK obtained for UV-DLIP, structures with a uniform and controllable geometrical pattern are observed.

#### 2.2.2 Conductive polymers

Electrically conductive polymers (CPs) contain conjugated *π*-electron systems that give them unique characteristics such as hybrid electronic-ionic conductivity. Its clearest representatives are polyaniline [14, 108] (PANI), polypyrrole (PPy) [109, 110] and polythiophene and their derivatives, and co-polymers such as poly(3,4-ethylenedioxythiophene) polystyrene sulfonate (PEDOT:PSS), that have been well-known for decades. Particularly, PANI has attracted considerable attention due to its favourable environmental stability, redox reversibility and electrical conductivity [110, 111]. Not only is the production of conductive bulk polymers technologically important, but also the fabrication of CPs surface patterns to produce devices such as biosensors, chemical sensors, microelectrodes, biomedical devices, artificial muscle and neural interfaces, battery electrodes, photoelectric cells, among others [14]. Although these polymers show a poor mechanical stability, they are generally stable as colloidal dispersions [112]. To achieve a conductive system with good mechanical stability it is necessary that these CPs form composite materials in combination with another material such as another polymer [113], glass [114] or metal [14]. Regarding the production of CPs films, this is generally done by classical chemical or electrochemical oxidative polymerization deposition, and less usually, photochemically, while patterning of conductive films was initially carried out using lithographic techniques or combined lithography and nanoimprinting techniques in multiple steps [115].

**Figure 11:**
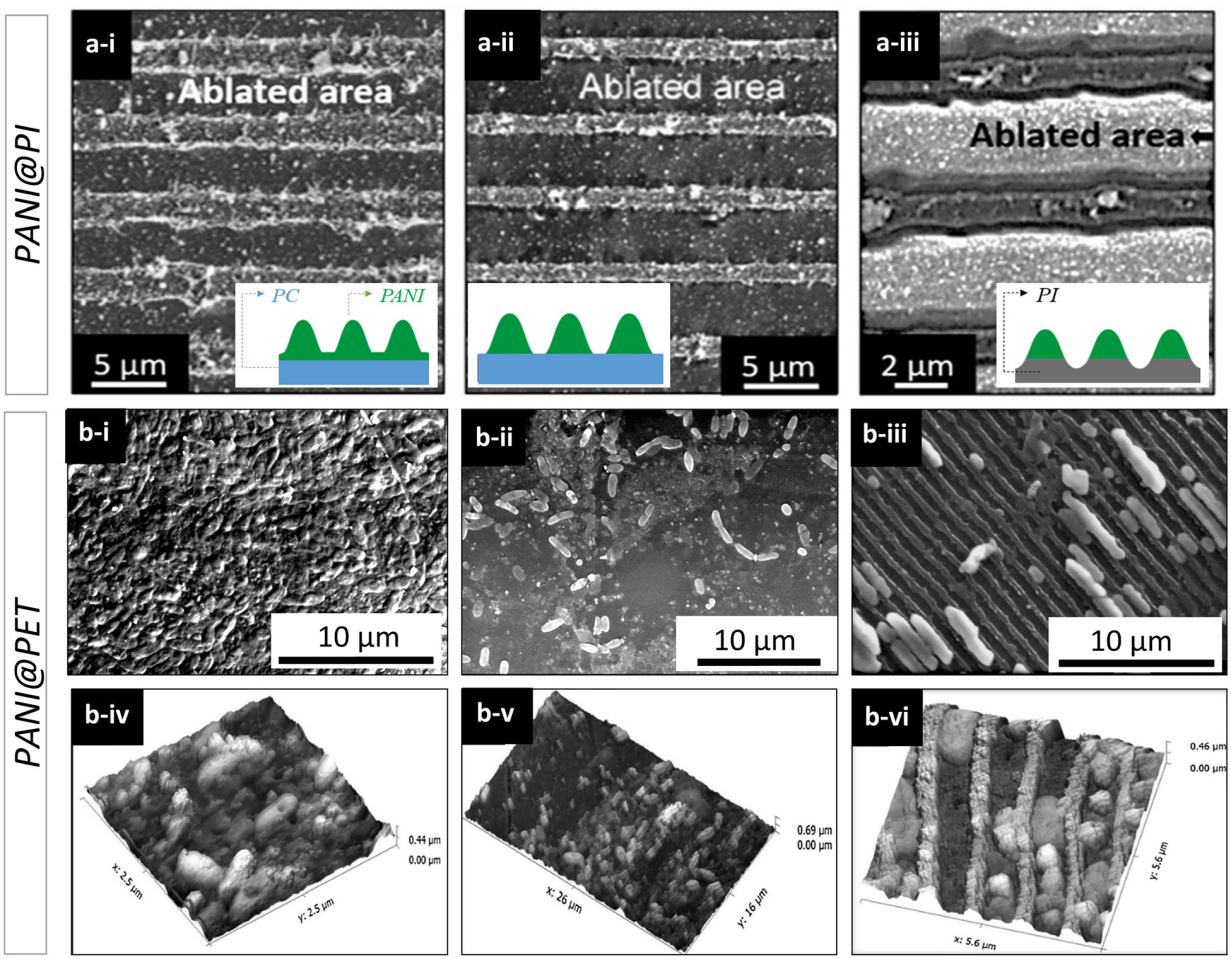
(upper row) SEM images of PANI@PC nanostructures supported at (a-i) laser fluence 325 mJ cm^−2^; (a-ii) laser fluence 174 mJ cm^−2^ and (a-iii) PANI@PI laser fluence 194 mJ cm^−2^ where the substrate is also ablated. The scheme bottom right the diagram indicates the type of material ablated; (middle and lower rows) SEM micrographs of the biofilm formed (grown for 24 h) on (b-i) PET, (b-ii) PANI@PET film and (b-iii) PET-PANI structured by DLIP and AFM micrographs of the bacteria grown for 24 h on (b-iv) PET, (b-v) PANI@PET and (b-vi) PANI@PET-DLIP. Figures adapted from ref. [116] (© 2007 WILEY-VCH Verlag GmbH & Co. KGaA) and ref. [78] (© 2016 Elsevier B.V.).

In 2007, Acevedo et al. [116] used DLIP to generate PANI nanoarrays using a UV laser source. In this research work it was shown how to obtain well-defined and controlled geometrical line-like patterns on PANI films chemically bonded on PC and PI using DLIP. The conductive structures were fabricated with a single laser pulse at 355 nm. The authors showed that increasing the laser fluence, the width of the PANI lines increased following three behaviours. In the first case (see Figure 11(a-i)), at low laser fluences (<174 mJ cm^−2^), the PANI film was ablated very locally at the positions of the interference maxima. As the laser fluence increased (Figure 11(a-ii), a larger amount of the laser energy exceeded the PANI threshold ablation fluence and larger ablation areas were achieved, regardless of the substrate. Finally, for the PANI@PI sample (Figure 11(a-iii)), irradiated with a fluence of 194 mJ cm^−2^, not only the PANI upper film was ablated but also the supporting polymer, PI. As a result, both the thickness and the width of the PANI lines decreased, achieving configurations where the PANI disappeared completely from the ablated zone giving rise to a surface with alternating conductive-insulator channels, produced at the minima and maxima of interference, respectively.

Acevedo and co-workers [59] investigated also this behaviour on different PANI substrates structured by DLIP. In another work they succeeded in generating a PANI@glass substrate with markedly anisotropic conductivity in the direction parallel to the patterned grooves. Following this work line, Acevedo and Lasagni [117] later developed a method to quantitatively measure the electrical conductivity of PANI films by indirectly sensing the specific electrical resistance of each pattern. The results indicated specific resistance values changing by an order of magnitude when comparing unstructured PANI films (PANI@glass) with those structured by DLIP (PANI@glass-DLIP). Furthermore, the electrical resistance was significantly different when measuring the film in the direction of the pattern and perpendicular to the pattern. However, the overall average specific conductivity across the untreated and patterned films was in the range of previously reported values in doped PANI (0.5–2.8 S cm^−1^). This represents further evidence of no significant electrochemical modification after DLIP treatment, paving the way for using these microelectrodes as electrochemical devices or as biomedical platforms.

The DLIP structured conductive polymers were further reported for the development of antimicrobial surfaces. Gallarato et al. [78] successfully fabricated PANI coated PET films structured by DLIP (PANI@PET) capable of decreasing biofilm formation of the bacterium *P*. *aeruginosa*. They noted that PANI@PET surfaces resulted in a significant inhibition of bacterial adhesion (more than 70%) and biofilm formation (more than 50%). Most remarkably, they also demonstrated that microstructuring PANI via DLIP synergistically increases the antimicrobial capacity of the PANI surface, decreasing bacterial adhesion by 97.5% and biofilm formation by 65%. Proving that there is a coactive effect due to the chemical and physico-chemical effects achieved on the original PET film due to multiple factors (chemistry, loading, mechanical properties and topography). As can be seen in Figure 11(b-i and ii) the appearance of *P*. *aeruginosa* bacteria growth on PET and PANI@PET showed a spherical shape forming a dense and homogeneously distributed biofilm along the entire surface in 24 h of culture. In contrast, on the DLIP treated sample (PANI@PET-DLIP) (Figure 11(b-iii)) the bacteria, acting in response to the topography, chose a preferred direction of growth, changed their morphology and modified the production of exopolysaccharides. Several scientists ascribed this as a strategy of the cell to survive when is exposed to stressful environmental conditions [11]. This work triggered further research on the generation of antimicrobial surfaces on PANI coated polymers structured by DLIP, as surface chemistry and micro/nanotopography of solid interfaces play an essential role in mediating the activity and adhesion of microorganisms, which is not yet fully elucidated in coated-polymers.

The DLIP structuring of polymer films coated with polypyrrole (PPy) was also reported [118]. For instance, PPy films deposited on inert polymeric substrates were irradiated by DLIP at 355 nm, so that only the conductive polymer film was patterned while the transparent support polymer remained unchanged. The period of the patterned grooves varied from 900 nm to 3.5 µm and the width of the PPy lines was adjusted by controlling the laser fluence. In addition, Fourier transform infrared (FTIR) spectroscopy and UV–VIS spectroscopy provided information to ensure that the chemical structure of PPy remained unaffected after the structuring process in the non-ablated regions. The electrical characterization showed a relatively low decrease of conductivity from 13 S cm^−1^ of the untreated film to 5.7 S cm^−1^ after the DLIP treatment. Hence, the patterned films can be used for sensor devices responsive to specific ions (e.g. gases such as ammonia) based on the electronic properties of the material. The researchers suggested that the small dimension of the conductive polymer lines achieved by DLIP would increase the response rate of the devices as microelectrodes due to two reasons. Firstly, the mass transport of charge will be enhanced because there is a longitudinal cylindrical diffusion and secondly the volume/surface area ratio is higher for structured films than for flat films [118]. In the same work Acevedo et al. [118] performed static water contact angle measurements to evaluate the wettability of structured PPy. The contact angle increased from 55° on flat PPy to about 75° on structured line-like PPy and 72° for pillar-like PPy (3.5 mm period). This phenomenon can be ascribed also to a Cassie-Baxter behaviour, in accordance with the above-mentioned experiments on patterned PU and observed on other conductive polymers [119].

#### 2.2.3 Cross-linked and block co-polymers

The use of DLIP has been reported not only on linear polymers but also for soft materials such as cross-linked polymers [120] or semi-interpenetrated networks [101]. For example, Molina et al. [121] used DLIP (355 nm, 10 ns pulse width) to create patterns on a cross-linked poly(N-isopropylacrylamide) hydrogels (PNIPAM) doped with a solution of tris (2,2′-bipyridine) ruthenium (II) dye (RBPY). In this case, the ablation effect involves a transfer of heat from the highly UV-absorbing dye to the transparent polymer. The PNIPAM are well-known biocompatible, thermoset and thermosensitive smart materials [120] able to undergo reversibly volume transitions between hydrophilic (swollen) and hydrophobic (unswollen) states at the lower critical solution temperature (LCST) of 32–32.5 °C [112]. The colorless (undoped) PNIPAM hydrogels did not form patterns when irradiated with 355 nm laser light, due to the negligible absorption of the polymer at that wavelength. The interesting feature of such materials is that when the surface is swollen in water, the DLIP texture disappeared. This is probably due to the expansion of the gels by swelling. However, it can be observed that when the hydrogel was dried by heating to a temperature above the LCST of the hydrogel, the patterned surface reappeared; thereby they reported the creation of a smart surface with triggered patterning due to swelling–deswelling cycles. A similar behaviour was observed on PNIPAM surfaces interpenetrated with PANI and structured by DLIP (PANI@PNIPAM) by Mulko et al. [122]. The presence of the light-absorbing PANI allowed the dry films to be patterned by DLIP at 355 nm, which in turn resulted in a surface nanofoam superimposed on a lines/groove pattern. In addition, the conductive PANI absorbed electromagnetic radiation (RF), which was converted to heat and induced the phase transition of PNIPAM. In this case, pattern activation was achieved by exposing the patterned surfaces swollen in water to the remote RF radiation inducing the hydrophilic-to-hydrophobic phase transition by increasing the temperature above the LCSTs of the material [122].

**Figure 12:**
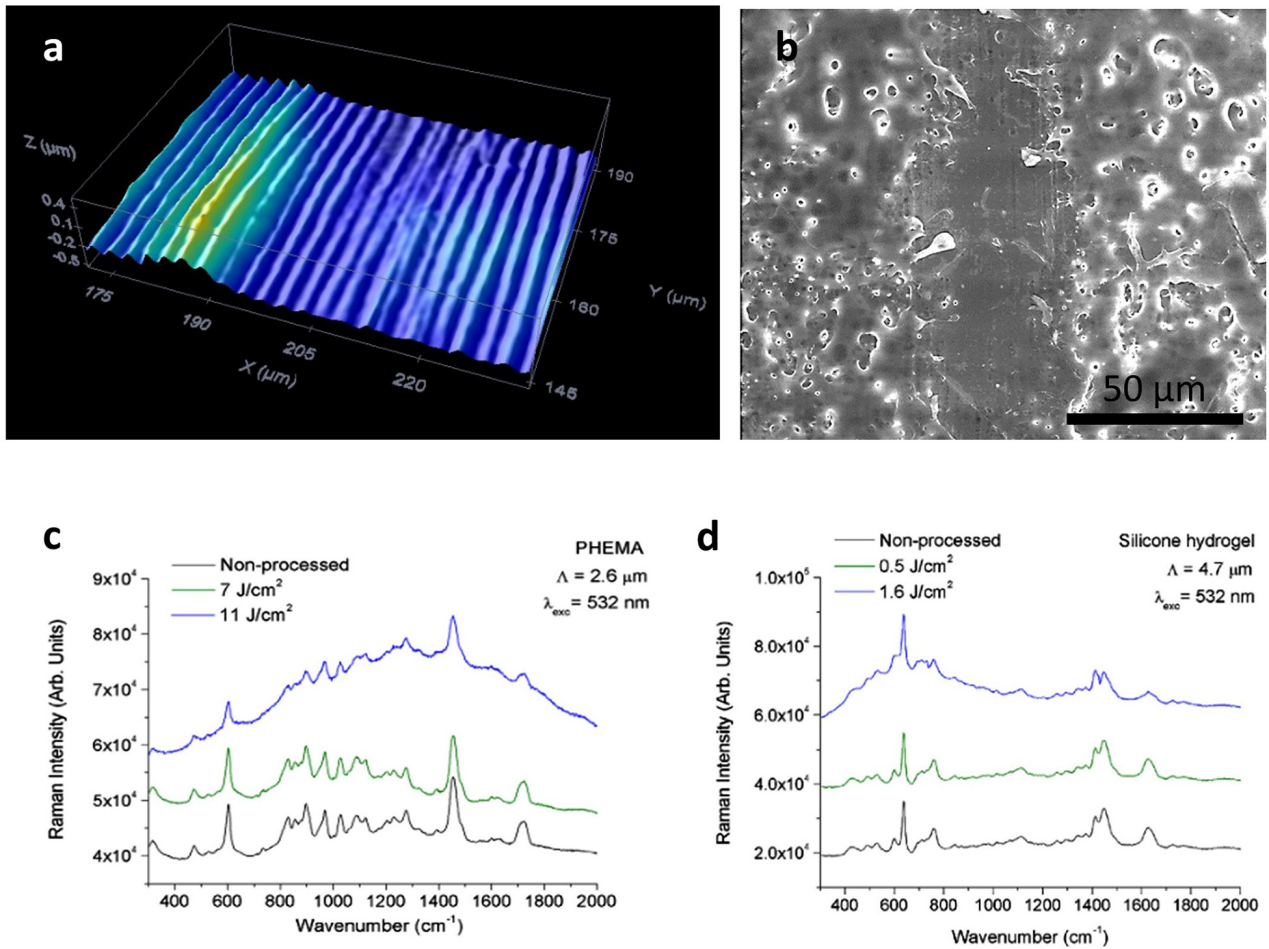
(a) Confocal image of a PHEMA sample structured (2.6 µm, 1 pulse, 7 J cm^−2^), (b) SEM micrograph PHEMA samples structured (2.6 µm, 1 pulse, 11 J cm^−2^), *µ*-Raman spectra of Micro-Raman spectra of (c) PHEMA sample in non-processed areas (black line) and in the DLIP structured regions with a spatial period of 2.6 μm at 7 J cm^−2^ (green line) and 11 J cm^−2^ (blue line) laser fluence (d) silicon hydrogel in non-processed areas (black line) and in the DLIP structured regions with a spatial period of 4.7 μm at 0.5 J cm^−2^ (green line) and 1.6 J cm^−2^ (blue line) laser fluence. Reproduced from ref. [123] with permission from Japan Laser Processing Society.

Sola et al. [123] also reported several works where line-like textures were patterned by DLIP in poly hydroxy ethyl methacrylate (PHEMA) films. In this case, PHEMA films were structured to modify their refractive index for ophthalmic purposes. The interference periods were fixed at 2.6 μm and 4.7 μm. The pattern characteristics were studied over a range of laser fluences between 0.5 J cm^−2^ and 17 J cm^−2^, and the number of pulses varied from 1 to 5. A confocal image of the structured zone at low fluence (7 J cm^−2^) is shown in Figure 12(a). At this working fluence the researchers reported that the laser-matter interaction process resulted in swelling of the polymer surface. This swelling phenomenon, as already mentioned, usually occurs in a short range of fluences. In contrast, at higher laser fluences, the structuring of the material occurred predominantly through ablation mechanisms. For example, as observed in the SEM micrograph in Figure 12(b), at a fluence of 11 J cm^−2^ for a spatial period of 2.6 µm, heat affected zones appeared as ablation or bubble-like areas because of the photothermal-mechanical nature of the laser–matter interaction process. The chemical composition and structural analyses of laser-treated polymers was investigated using Raman spectroscopy [123]. This is a powerful technique to explore polymeric films treated by laser as long as no increase in the fluorescence background (usually assigned to thermal decomposition of short-chain organic molecules) arises, which may screen out key characterization bands. Unfortunately, this type of behaviour is quite common in polymers such as PC, PEEK and PI treated by nanosecond lasers [94]. Nevertheless, Sola et al. [123] reported DLIP-structured *µ*-Raman spectra at 532 nm for PHEMA and silicone hydrogels. The Raman spectra of the DLIP structured areas processed at high fluences (Figure 12(c) and (d) – blue line) showed variations compared to those corresponding to the unprocessed samples (Figure 12(c) and (d) – black line). In particular, in the PHEMA sample processed at high laser fluence (11 J cm^−2^) there was a strong decrease in the peak intensity of several bands (473 cm^−1^, 604 cm^−1^, 830 cm^−1^, 897 cm^−1^, 1089 cm^−1^ and 1718 cm^−1^), while other bands disappear (734 cm^−1^, 1204 cm^−1^, 1230 cm^−1^). A similar behaviour was detected for the DLIP-modified silicone hydrogel and shown in Figure 12. In contrast, at low intensity, laser structuring was not accompanied by significant changes in the *µ*-Raman spectra, hence the polymer structure remained almost unchanged after laser irradiation.

Acevedo et al. [58] reported the structure of a block co-polymer where the swelling phenomenon (already described in Section 2.2.1) is also observed. They presented the fabrication of advanced architectures using DLIP at 266 nm in poly (glycidyl methacrylate-co-styrene) with four different percentages of styrene P(GMA-%S). It was shown the transition from a uniform line-like structure with interference ablation to a less uniform and more disordered structure where the swelling phenomenon underlies. The variables studied being not only the laser fluence used, as is commonly observed, but the percentage of styrene present in the co-polymer. As expected, the surface of the PS homopolymer was ablated at the position of maximum interference but in the P(GMA-%S) the surfaces were ablated and swollen in the regions of maximum interference. The styrene content in the polymer absorbed the laser energy giving rise to photothermally ablated regions or promoting chemical decomposition of the acrylate units or polymer segments.

## 3 DLIP processing of ceramics and semiconductors

Ceramics are characterized by a high hardness, corrosion resistance, chemical stability, wear resistance and capability to sustain high strength at higher temperature than polymers and metals. Moreover, ceramics can be insulators, semiconductors or conductors and can be used also as biocompatible materials. However, their characteristic brittleness makes ceramics hard to machine, increasing the cost of the final part, especially during the finishing step [124]. Conventional mechanical machining methods turned out to be inefficient to treat ceramics due to tool wear, vibrations, and long process times. Alternatively, non-contact techniques that avoid the use of hard tools, such as ultrasonic machining, electric discharge machining, direct laser writing, plasma machining, abrasive water jet machining or electron beam machining, have entered the market in recent decades. Nevertheless, when it comes to large-area surface patterning with high-resolution down to the sub-µm scale and with high flexibility, DLIP appears as a promising industrial solution. In the following sections, an overview of recent progress towards surface micropatterning of ceramics by DLIP is given.

### 3.1 Bioceramics

**Figure 13:**
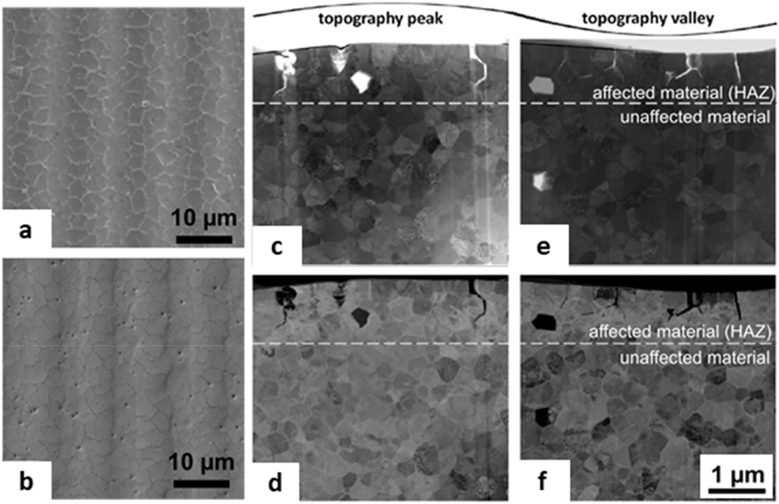
Comparison of DLIP processing on zirconia with two different wavelengths: (a) 532 nm and (b) 355 nm. STEM images of FIB-cut lamella across a ridge peak (c) and (d) and valley (e) and (f). Bright field images are shown in (c) and (d), whereas dark field images in (d) and (f). Figures adapted from ref. [128] (© 2017 Elsevier B.V.).

Ceramics have been used as biomaterials for decades not only due to their non-cytotoxicity and resistance to corrosion, but also because an important constituent of bones are ceramic materials [124]. Particularly, alumina and zirconia have been widely used for dental implants and for prosthesis due to the excellent biocompatibility compared to other ceramics. Roughening the surface of these materials can enhance their adherence to surrounding materials, such as cements, dental tissues like dentin and enamel or underlying metallic substrates, and improve osseointegration [125]. Several studies have proved the feasibility of structuring zirconia by ns-DLIP employing two overlapping beams to pattern periodic grooves with spatial periods between 1 µm and 15 µm and aspect ratios up to 0.6 [126]. Roitero et al. [127] performed a thorough microstructural and chemical analysis on ns-DLIP treated yttria-stabilized zirconia yielding new insights into the material modifications after laser structuring. From SEM analysis combined with FIB machining, they observed a network of interconnecting cracks on the treated zirconia that was independent of the chosen laser wavelength, 532 nm (Figure 13(a)) and 355 nm (Figure 13(b)), and texture periodicity. They found also that the heat-affected zone (HAZ) extended only 1 µm below the surface and it could be split into two layers as shown in the STEM cross cuts of Figure 13(c)–(f). The top layer, with a thickness of ∼300 nm, was mainly formed by recast material characterized by columnar grains oriented perpendicular to the surface. The bottom layer was composed of isotropic grains, which were highly deformed due to high dislocation density and twinning. Cracks homogenously distributed were observed both at the peak (Figure 13(c) and (d)) and valley (Figure 13(e) and (f)) of the microtexture, crossing through all the HAZ along the grain boundaries.

Laser structuring also induced structural transformations, resulting in the appearance of a monoclinic phase co-existing with the tetragonal phase which was also observed in the untreated sample. Chemical analysis by X-ray photoelectron spectroscopy (XPS) revealed that the composition remained approximately constant after the laser step. The pattern formation on zirconia was mainly explained by thermal mechanisms due to the long pulse duration in the ns range. The absorbed laser energy led to melting and evaporation, but also accompanied by liquid flow attributed to the large surface tension gradients [127, 128]. Previous studies from the same research group have shown an increase of fracture toughness, measured as flexural strength, of up to 50% in yttria-stabilized zirconia. The presence of high compressive stress on the surface combined with a periodic distribution of pore sizes and grain refinements down to the nanometer scale resulted in an increase of the force needed to bring the surface into a tensile stress and to trigger crack formation, which ultimately leads to an increase in the flexural strength [129, 130]. Similar experiments were conducted on alumina that led also to an increase in flexural strength. These findings have the potential to increase the resistance and lifetime of dental restorative materials based on zirconia and alumina.

**Figure 14:**
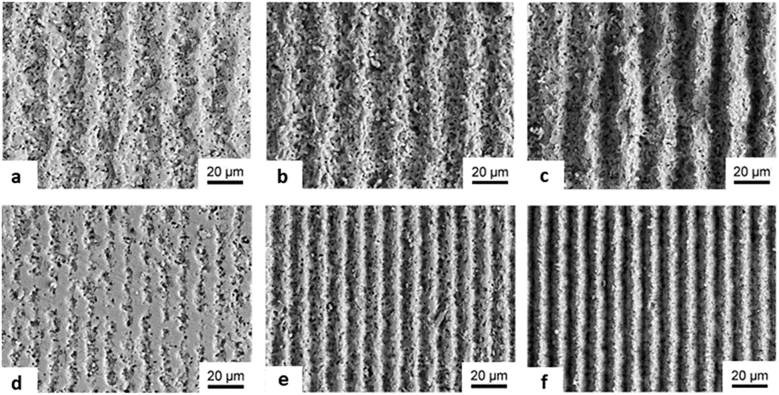
SEM-micrographs of periodic line-like patterns fabricated on hydroxyapatite with 355 nm laser radiation, 20 µm period and 1.2 J cm^−2^ of laser fluence with (a) one, (b) 10 and (c) 50 pulses and with 266 radiation, 10 µm period, fluence of 0.6 J cm^−2^ with (d) one, (e) 10 and (f) 50 pulses. Figures adapted from ref. [134] (© 2011 Elsevier B.V).

Due to its outstanding cytocompatibility, synthetic hydroxyapatite (HAp), Ca_5_(PO_4_)_3_(OH), is one of the most used bioceramics for artificial bone replacement that can successfully mimic bone tissues [131, 132]. Furthermore, engineering the surface texture of HAp can enhance the osseointegration of the implant and induce cell organization [133]. Berger et al. [134] have conducted a detailed analysis of the influence of ns-DLIP process parameters on the resulting surface morphology of laser treated HAp. Line-like and cross-like (pillars) textures with spatial periods of 10 µm and 20 µm were produced by overlapping two laser beams. The experiments were conducted with two different wavelengths in the UV spectrum, namely 266 nm and 355 nm. The authors found significant differences in the surface morphology upon processing HAp with either 355 nm or 266 nm radiation, as shown in the SEM images of Figure 14(a)–(f), respectively. In the latter case, the ablation process was mainly dictated by photo-thermal mechanisms followed by melting. Due to the low thermal conductivity of HAp, large temperature gradients (2000–3000 K) were established between maxima and minima of the interference pattern on the sample resulting in large thermal stresses. Additionally, such stresses were boosted by the presence of pores in HAp that disrupt the heat flow, and that ultimately can destroy the repetitive textures when large accumulated fluences are applied on HAp or when short spatial periods (<10 µm) are targeted. In turn, the ablation process of HAp with 266 nm radiation has not only a contribution from photothermal effects but also from photochemical reactions. That is, after laser irradiation, the excited material decomposes into several constituents without a significant temperature rise. Interestingly, even at high accumulated fluences, XPS analysis confirmed that no significant modifications in the chemical composition of the HAp surfaces were produced after the DLIP process.

### 3.2 Semiconductors and oxides for electronics

Transparent conductive oxides (TCOs) are widely used in sensors and optoelectronic devices based on thin film semiconductors [135]. The outstanding combination of optical transmittance in the visible spectrum, relatively low electrical resistance, chemical inertness and mechanical strength, has made TCOs a key component in thin film solar cells, OLEDs, touch screens and LCD displays. A surface pattern on the TCO surface can be engineered to manage light conveniently to, for example, trap more light in a solar cell or to couple the light generated in an OLED into the outer medium as well as to tailor the electrical resistivity.

DLIP processing on doped and undoped zinc oxide (ZnO) films has been extensively investigated in the last decade, especially using ns-laser sources emitting in the UV spectrum. Nakamura and co-workers have patterned line-like textures and square arrays of holes on ZnO thin films by interfering four UV (355 nm) beams using a 4f interference configuration [136]. Then, they grew periodic ZnO nanocrystals and nanowires on the DLIP structured films by nanoparticle-assisted pulsed laser deposition (NAPLD) [137].

**Figure 15:**
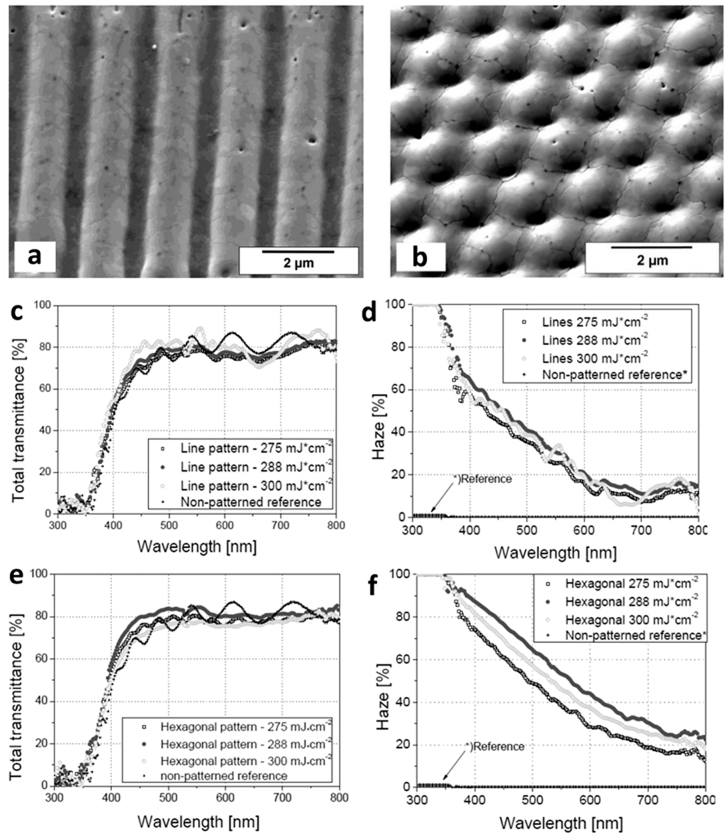
SEM micrographs of AZO films structured by DLIP with a (a) line-like pattern and (b) hexagonal pillar-like array. Total transmittance and haze factor of the hexagonal ((c) and (d), respectively) and line patterns ((e) and (f), respectively). Figures adapted from ref. [143] (© 2013 Elsevier B.V).

As doped ZnO films are frequently used in optoelectronic applications, textures with targeted periodicities in the range 0.5–1.5 µm have been mainly produced by DLIP to enable a strong interaction between visible light and the periodic surface grating. A detailed morphological analysis was realized by Eckhardt et al. [138] on DLIP-treated aluminum doped ZnO (AZO) films using ns pulses. Figure 15 shows exemplarily a (a) line-like and (b) hexagonal pillar-like texture reported in that work. They found that thermal-induced surface tension gradients were the main mechanism by which molten material was dragged from the intensity maxima positions to the minima, as was also observed in ns-DLIP processing of ZnO:B [139]. During the cooling phase, the material crystallized forming large grains but also a noticeable distribution of cracks, in accordance with the findings by Parellada-Monreal et al. [140] and Knüttel et al. [141]. On the contrary, texture formation on AZO films by ps-DLIP presented a significant contribution of ablation and a smaller amount of molten material compared to ns-pulsed processing. This can be attributed to the shorter thermal diffusion length below 20 nm [142] compared to a thermal diffusion length of ∼1 µm for ns-pulses, which leads to very localized heating and temperature rise above the vaporization temperature of AZO. Optical characterization of the DLIP structured ZnO films demonstrated the ability of such textures to scatter and diffract light very efficiently, as shown in the global transmittance and haze factor measurements of Figure 15(c) and (e) and Figure 15(d) and (f), respectively [143]. This enhanced optical management can be exploited to boost the efficiency of thin film solar cells [144]. In fact, DLIP-patterned AZO have been used as substrate in a-Si:H/µc-Si:H tandem solar cells, showing an increase in photocurrent by 20% [142] and also as substrate in OLEDs with an enhanced external quantum efficiency of more than 25% [145].

**Figure 16:**
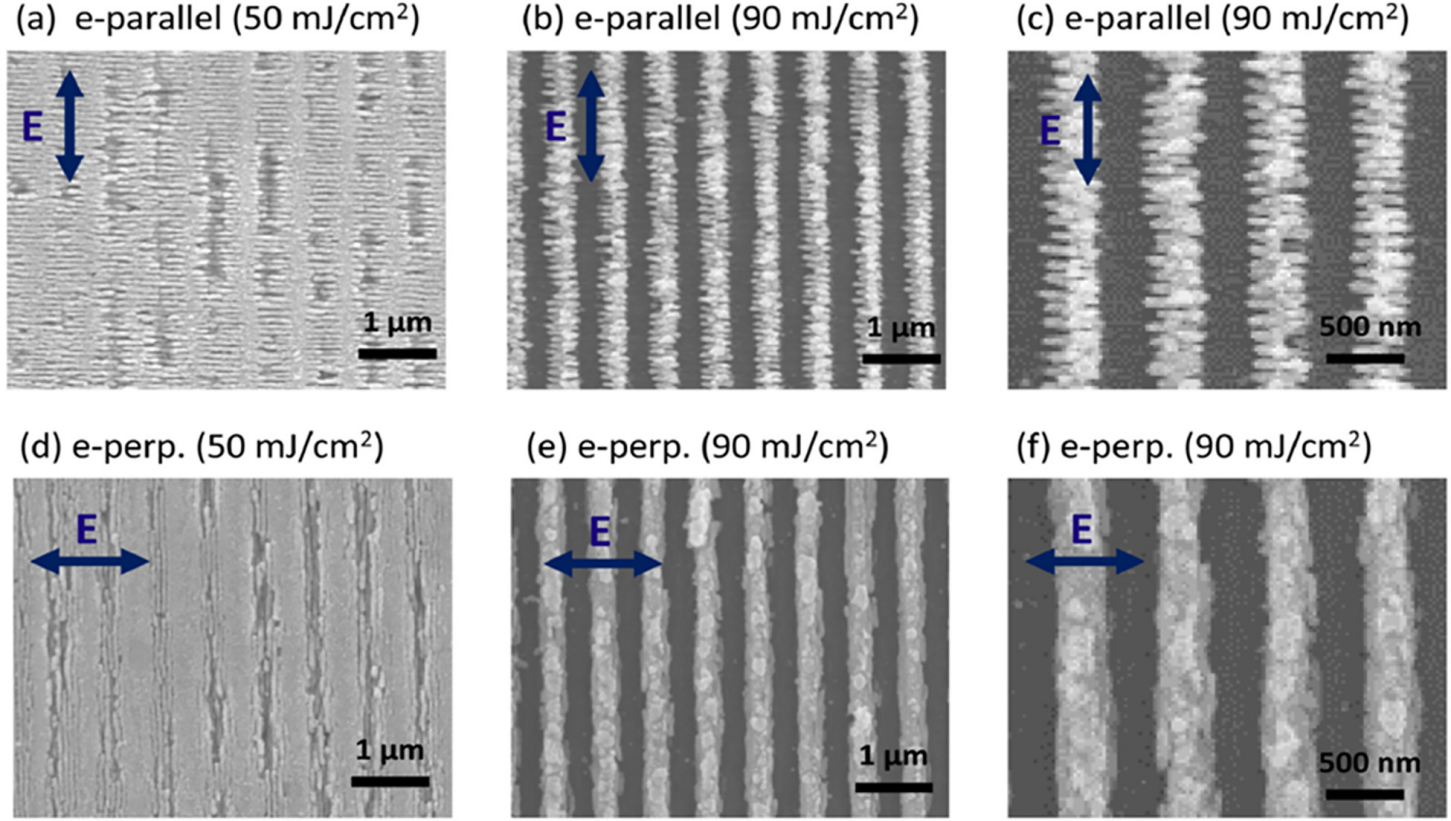
ITO films structured by ps-DLIP with different polarization directions (as indicated by the arrows) and fluences, which in turn, influenced the alignment and dimensions of LIPSS. Figure reproduced from ref. [146] (© 2019 Optical Society of America).

Two-beam DLIP was also successfully used to patten line-like textures on indium tin oxide (ITO) films with ps-pulses at a wavelength of 355 nm [146]. In this case, it was explored the feasibility of producing hierarchical nanotextures by combining the DLIP features with LIPSS, as shown in the SEM images of Figure 16 for different polarization directions and laser fluence. The resulting multiscaled patterns exhibited a strong anisotropic electrical resistance characterized by a ratio between the longitudinal and transverse resistance of more than 50,000. Besides, InO_*x*_ thin films were patterned by overlapping two beams from a ns-pulsed KrF excimer laser source emitting UV (248 nm) light [147]. Here, the authors reported two characteristic ablation regimes which can be correlated to the initial electrical properties of the material. Namely, a transition from a semiconducting behaviour to a conducting characteristic as a function of the UV exposure was observed. As a consequence, in the early stages of irradiation the material ablation can be attributed to photochemical mechanisms, but as the cumulated fluence increased, thermal ablation was triggered increasing the ablation rate and influencing the quality of the final topography. The fabricated relief diffraction gratings with a spatial period of 610 nm were then applied for the fabrication of Bragg grating mirrors in InO_*x*_ thin film overlaid waveguides, which can be useful for filtering devices in optical communication applications.

As the mainstream semiconductor for microelectronics and photovoltaics, silicon has become a ubiquitous material in our daily life. Laser processing of silicon wafers and thin films has become common practice in the last decade for mainly applications such as selective crystallization [148], vias drilling for sensors [149], laser scribing for solar modules interconnections [150], and for realizing local contact openings at the rear side of solar cells [151]. Also, fs-laser surface texturization by LIPSS has been used for enhancing the absorption of solar cells by reducing the reflectance of sunlight [152].

DLIP patterning with ns pulses has been extensively employed to modify the topography of Si wafers [153]. Due to the high evaporation point of Si, it is commonly agreed that Marangoni convection is the dominant mechanism for the formation of the repetitive microstructures in the ns-pulse duration regime [154, 155]. Peláez et al. [154] overlapped two beams from an ArF excimer laser with a wavelength of 193 nm at a pulse duration of 20 ns on Si to produce line-like textures with a period of 1.7 µm. They performed a sound process parameters screening, where fluence in the range 0.63–1.0 J cm^−2^ and number of applied pulses in the range 1–5000 were swept. They correlated the resulting grooves morphology, i.e. height and FWHM, with the process parameter and explained their formation by an analytical model based on thermocapillary forces and surface tension gradients (Marangoni convection), ruling out significant effects from material vaporization. Also periodic micropillars arrays were fabricated by overlapping either three or four beams from a ns-laser source. The structure formation was achieved by the same melting, material flow and resolidification mechanisms described above [155]. DLIP patterned microcones on Si allowed for a strong reduction of reflectivity below 10% in the visible spectrum [153], which is significantly lower than the reflectivity of 30–40% measured in polished Si over the same spectral range [156]. Although these results are promising for producing silicon solar cells with antireflective properties, the possible significant crystallographic or chemical modifications in the heat affected zone may deteriorate the electronic properties on the silicon surface as well as along several µm below the surface. Therefore, more investigations need to be done to assess the feasibility of using this laser-based approach for improving the overall performance of Si solar cells.

**Figure 17:**
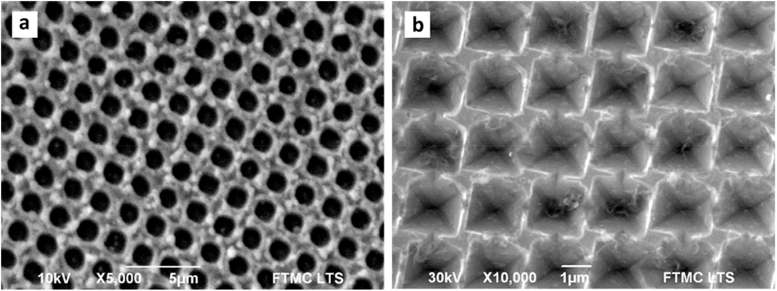
SEM images of a silicon wafer structured by (a) four-beam DLIP and (b) by post-laser chemical etching with controlled anisotropy. Reproduced from ref. [159] (© 2014 SPIE).

Also, DLIP based on fs-laser sources was used to pattern the surface of Si wafers. Given the ultra-short pulse (USP) duration of 560 fs, at a wavelength of 1030 nm, Oliveira et al. [157] proposed to use a Michelson interferometer to overlap two beams on a Si wafer. Thereby, gratings with a spatial period of 720 nm were produced by material ablation at the intensity maxima positions. Another configuration able to yield an interference pattern by overlapping USP is based on a Schwarzschild objective, as reported by Ihlemann and co-workers [158]. With this setup they could pattern linear gratings on silicon using a UV excimer laser with pulse durations from 500 fs to 50 ps. Voisiat et al. [159] have used a diffractive optical element (DOE) and two lenses arranged in a 4*f* configuration to overlap four and six fs-beams on Si. The resulting square and hexagonal arrays of microholes were then beneficial for inducing the formation of inverted micropyramids by anisotropic wet chemical etching. For instance, Figure 17 shows a square array of hole patterned with four-beam DLIP (a) and the resulting inverted micropyramid array after the chemical etching (b). Due to the applied fs-pulses, the heat affect zone encompasses only ∼10 nm below the Si surface avoiding a negative impact on the electronic properties. Although more investigations are needed, this approach can control the reflectivity of Si solar cells without comprising their electrical performance.

Other semiconductors were structured by DLIP too [19]. For instance, Ihlemann’s group reported the fabrication of complex periodic micro and sub-microstructures on GaN substrates by phase-controlled multiple beam interferometric projection [34]. In their setup, three linear phase gratings, rotated by 30° to each other, were used as DOE and the first diffraction orders from each of the gratings were allowed to interfere. The laser source was an excimer laser emitting 500 fs-pulses at a wavelength of 248 nm.

**Figure 18:**
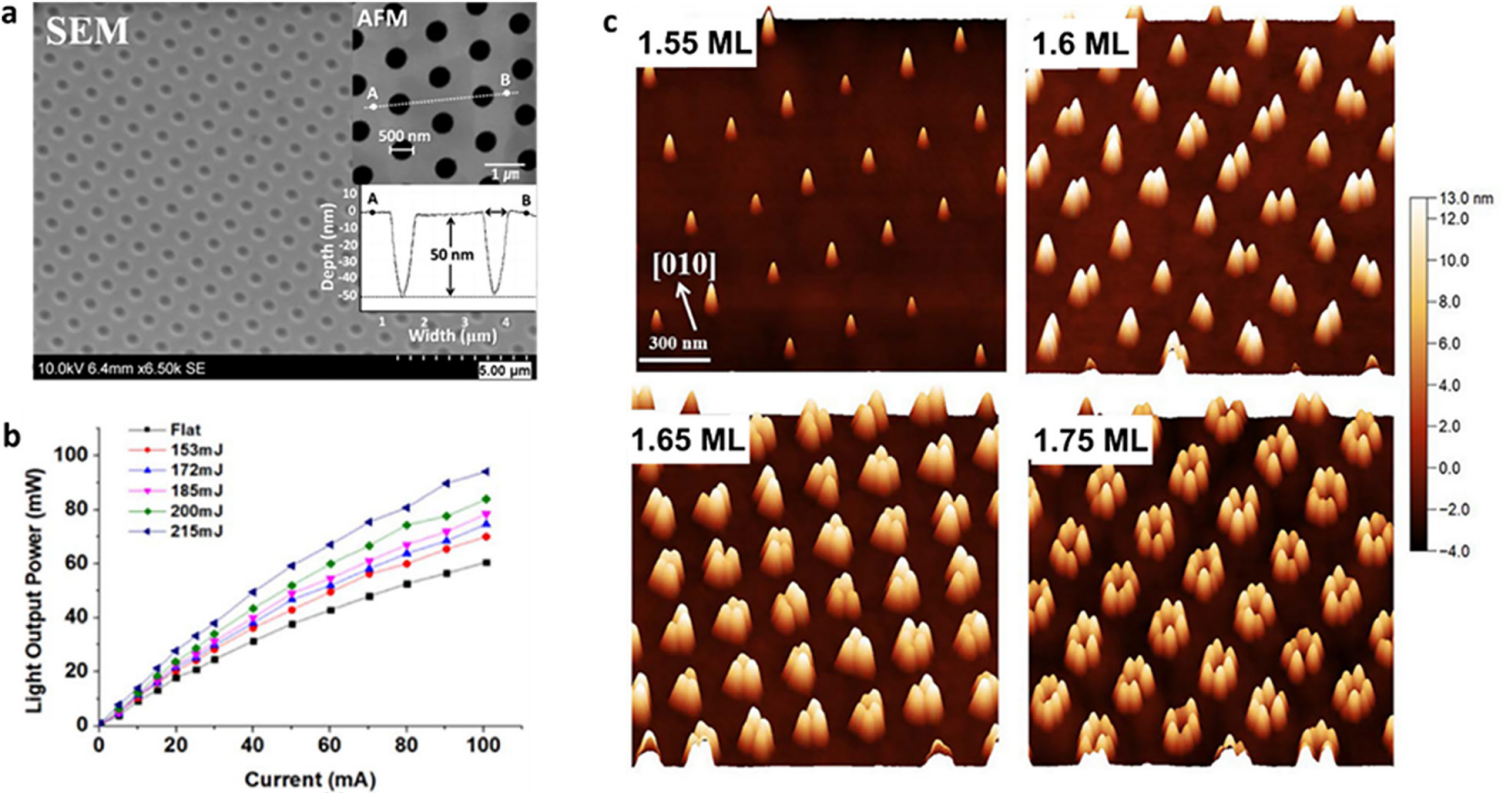
(a) Topography images of DLIP-treated LED based on GaN. (b) Optoelectronic characterization of structured LEDs, showing an increase in the performance. (c) AFM images of quantum dot arrays with different coverage of InAs monolayers (ML). Figures reproduced from ref. [160–162] (© 2014 AIP Publishing LLC, © 2017 Elsevier B.V, Licensed under a Creative Commons Attribution).

LEDs based on epitaxy-grown GaN heterostructures were irradiated by overlapping three UV ns-pulsed beams, resulting in hexagonal arrays of sub-micron sized holes [160, 161] as shown in Figure 18(a). Although Kim et al. [160] detected an increase in the series resistance of the laser-patterned LED, they attributed it to the locally reduced thickness of the top layer and not to a deteriorated ohmic contact. The grating-like topography of the DLIP-structured GaN LEDs yielded a significant increase in the light outcoupling efficiency by more than 55% compared to the reference device (Figure 18(b)) [161].

Quantum dots (QD) arrays were grown by a combination of self-assembled epitaxial growth of GaAs/AlGaAs and InAs/GaAs heterostructures combined with an *in-situ* DLIP process step [162, 163]. In this case, a beam from a ns-laser source emitting pulses at a wavelength of 355 nm was divided into four beams by beam splitters and directed into the molecular beam epitaxy (MBE) vacuum chamber through transparent viewports. As the UV light is strongly absorbed by the InAs monolayer (ML), strong lateral thermal gradients arose between the interference maxima and minima position, driving the atoms towards the cold areas and forming periodic arrays of nanoislands. Varying the MBE deposition conditions, the number of InAs monolayers covering the substrate and the laser polarization, Wang et al. [164] obtained arrays of QD and nanoislands with different geometrical shapes and aspect ratios, with a fixed spatial period of 300 nm, as shown in the AFM images of Figure 18(c).

Recently the active material of an antimony(III) sulfide (Sb_2_S_3_) based solar cell was patterned with a line-like texture with a spatial period of 1.2 µm using a ns-laser emitting pulses at 532 nm [165]. Interestingly, the researchers observed a double enhancement effect on the performance of the solar cells. One the one hand, the periodic microtexture increased in the haze factor dramatically, which increased the absorption and short circuit current. On the other hand, the Sb_2_S_3_ film recrystallized forming smaller grains than the as-deposited film, which led to an enhancement in the recombination and transport properties resulting in an increased fill factor and open circuit voltage.

In another work, spray-deposited TiO2 was processed with ns-DLIP (1053 nm) to achieve a cross-like texture with a period of 8.5 µm [166]. The film underwent crystallographic transformations from anatase to rutile phase at high number of pulses. Besides, the authors studied the photochemical, wettability and optical properties of the structured TiO_2_ and found a significantly increased photocatalytic behaviour.

### 3.3 Dielectrics

Highly transparent dielectrics like glasses, diamond or sapphire can be directly patterned using lasers by either linear absorption of UV radiation or by non-linear absorption mechanisms triggered by high-energy USP in the visible or IR spectrum. The latter phenomenon, which was described by many studies published elsewhere [26], consists basically in multi-photon ionization upon irradiating the material with USP, so that free electrons are generated in the conduction band. This step can then be followed by an avalanche impact ionization process, whereby the free electrons with high kinetic energy can transfer part of their energy to valence band electrons, which in turn can cross the band gap to the conduction band. Eventually, a sufficiently high number of excited electrons exceeding a given threshold can lead to material breakdown and macroscopic ablation [167, 168].

Particularly, mostly non-linear absorption was exploited in DLIP manufacturing for structuring transparent dielectrics. At the beginning of this century, a few pioneering works were reported by Kawamura and co-workers [169, 170] dealing with interference patterning on dielectrics with fs-pulses in the NIR spectrum. They have succeeded in engraving line-like patterns with a single laser shot on diamond [171], silica glass [172], lithium fluoride [173] and sapphire [169], among other transparent materials. Following a similar approach, Han et al. [174] have patterned silica glass with hexagonal arrays of holes by overlapping three fs-beams. They explored different symmetric and asymmetric hexagonal arrangements by adjusting the overlapping angles between the beams. In their study, the ablation mechanism was attributed to shock waves generated after the multiphoton ionization and avalanche ionization, which induced plasma expansion, phase explosion, melting and ultimately lead to local ablation.

**Figure 19:**
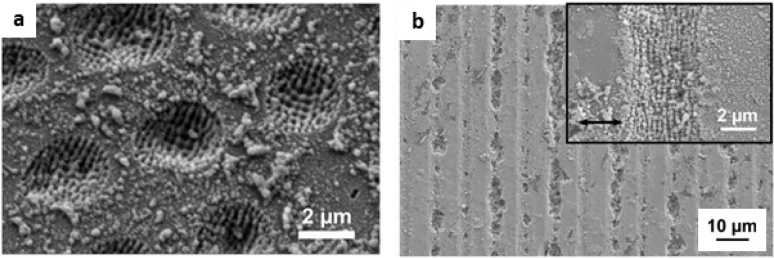
Microstructured sodalime glass by ps-laser radiation in the visible (532 nm) with (a) four and (b) two overlapping beams. In both cases LIPSS can be seen on the glass surface. The inset in (b) shows LIPSS aligned perpendicular to the laser polarization direction (double arrow). Reproduced from ref. [176] (Licensed under a Creative Commons Attribution).

LIPSS were also observed after DLIP processing of dielectrics using USP. In fact, Alamri et al. [95] have analyzed the interaction of DLIP patterning with the occurrence of LIPSS on sapphire by using linearly polarized fs-radiation and different fluence doses. In agreement with other sources [175], they found that the absorption of IR laser light (1030 nm) in sapphire was mainly governed by multiphoton ionization. Although low spatial frequency LIPSS perpendicular to the DLIP grooves were observed on sapphire, the process window resulted very narrow and strongly dependent on the presence of surface defects. A recent publication from the same group reported the feasibility of structuring float glass by two- and four-beam ps-DLIP using visible radiation (532 nm), varying the spatial period and fluence dose [176]. The line- and hole-like textures carved on the glass were attributed to non-linear absorption as well. Also, low spatial frequency LIPSS perpendicular to the radiation electric field were observed at the positions of the interference maxima in both types of textures, as can be seen in the SEM images of Figure 19.

DLIP ablation induced by lineal absorption in several dielectrics was also observed. For instance, to increase the absorbance and machinability of borosilicate glasses, Ag- and Er/Yb-dopants were introduced and structured with UV radiation [51, 177, 178]. In this way, high quality relief gratings with spatial periods down to the sub-micron scale were achieved. In the same direction, Beinhorn et al. [179] reported the fabrication of sub-micron surface gratings on Ta_2_O_5_ waveguide layers by overlapping two beams from a fs-pulsed excimer (248 nm) laser source using a Schwarzschild objective. Moreover, due to the utilization of USP in the fs-range, deep structures with remarkable quality were patterned.

In another study reported by Vlcek et al., thin films of As and Ge based glasses from binary (As-S, As-Se, Ge-S) and ternary systems (Ge-X-S, where X stands for As, Sb, Ga, In) were grown on glass slides and then were irradiated with two overlapping beams emitted from a pulsed KrF excimer laser at a wavelength of 248 nm and pulse duration of 20 ns [180]. The produced line-like topography featured a spatial period of 540 nm with depth in the range of 100–300 nm, depending on the sample composition and number of applied pulses. The mechanism of the relief grating formation could be explained by a major contribution from local melting and glass softening followed by mass transport to the positions of interference minima. Even though pulses with a duration of 20 ns were shot on the surface, local ablation could not be discarded due to the high absorption of UV radiation (248 nm) in these materials. Finally, the authors evaluated the performance of the structured films as resonant waveguide gratings in the IR spectrum (1550 nm).

In a recent work, thin films of lithium niobate (LiNbO_3_) were patterned by two-beams DLIP using a solid state frequency quintupled laser emitting at 213 nm with a pulse duration of 7 ns. The fabricated relief gratings with sub-micron spatial periods were successfully used as grating couplers for coupling NIR light to lithium niobate-on-insulator ridge waveguides [181].

## 4 Special cases

This section discusses DLIP processing of carbonaceous materials and their derivatives. In a series of linked papers Roch et al. [182], [183], [184] studied the influence of surface topography on the tribological properties of DLIP-structured tetrahedral amorphous carbon (ta-C). In the first paper [183], a 355 nm pulsed UV laser with two- and three-beam interference configurations was used to fabricate periodic arrays in the form of dots and lines. One of the major results of this study was the appearance of graphitization of the film during the laser processing, which occurs at varying fluence thresholds (47–74 mJ cm^−2^) depending on the number of laser pulses (up to 30). Their tribological behaviour was evaluated by reciprocal sliding tests by the ball-on-disk method under non-lubricated conditions. Because of the reduction of the contact area due to topographical effects in combination with the partial graphitization of the film, the coefficient of friction (COF) was reduced by approximately 30% in the patterned ta-C films compared to the unstructured reference.

**Figure 20:**
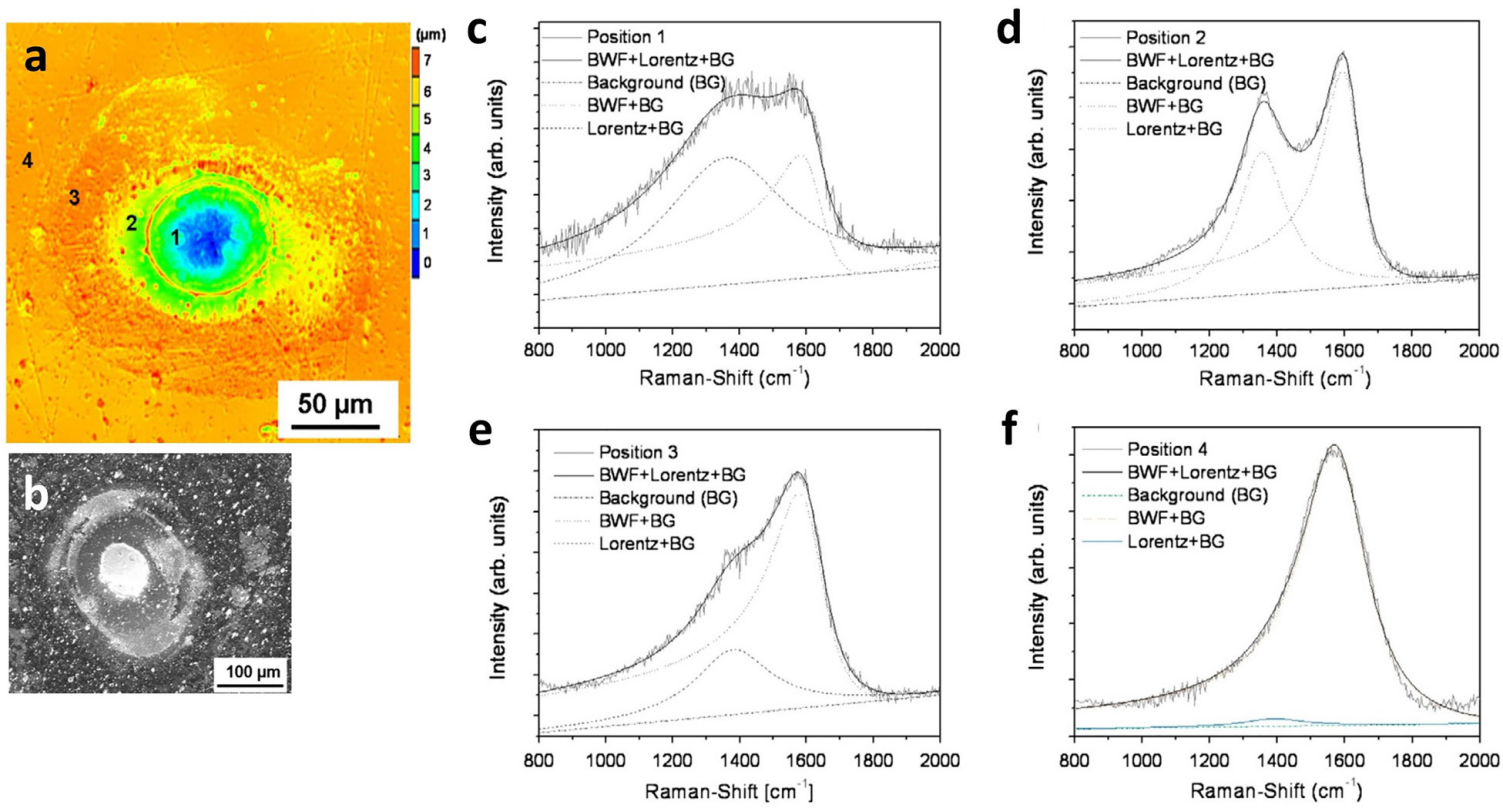
(a) and (b) LSM image and SEM image of the ablated ta-C film (fluence of 410 mJ cm^−2^, *N* = 30). The displayed positions correspond to: (1) the molten substrate, (2) partially ablated ta-C, (3) graphitized ta-C and (4) unmodified ta-C; (c)–(f) Raman spectra at the four different positions depicting the carbon morphology. Figures reproduced from ref. [183] (© 2013 Elsevier B.V.).

For characterizing the structured films, the authors performed SEM imaging and Raman spectroscopy. In the latter, different zones concentric to a central spot (following the Gaussian shape of the holographic pixel) were analyzed (see Figure 20(a) and (b)). Four distinct classes of ta-C were observed, which were monitored according to the well-known ratio of intensities of the peaks of the D-(disordered carbon band, 1350 cm^−1^) and graphitic band, G-(1580–1630 cm^−1^). In the central region of the laser spot (Figure 20(c)), the irradiated energy was high enough to soften the ta-C film and also to melt and soften the steel substrate (characterized by two broad and low D- and G-peaks in the Raman spectrum). Around the molten steel, in the form of a ring, a second region can be identified in which the carbon layer was partially ablated (Figure 20(d)). A third region can be distinguished in which the ta-C was locally graphitized (splitting of D- and G-peaks and increase of D-peak). Finally, a fourth region (Figure 20(f)) where the ta-C remains unmodified (dominated by a broad peak resulting from the superposition of peaks G and D). These findings are consistent with those from other publication of the same research group [185], where they reported that, depending on the laser energy density used for DLIP, the tetrahedral carbon film can graphitize or crystallize locally at the positions of the interference maxima. Complementary, in a third paper the authors [184] reported that depending on the structuring period, the COF increases or decreases compared to the reference samples. For small interference periods (approximately 2 μm), induced surface topographies may act as traps for wear particles, whereby the reduction of friction could be attributed to an increase of the contact surface and a concomitant increase of adhesive interactions. Alternatively, over periods of 3–10 µm dominant graphitization effects at the positions of the interference maxima were observed, which induced changes in the hardness of the material and thus increasing the COF.

**Figure 21:**
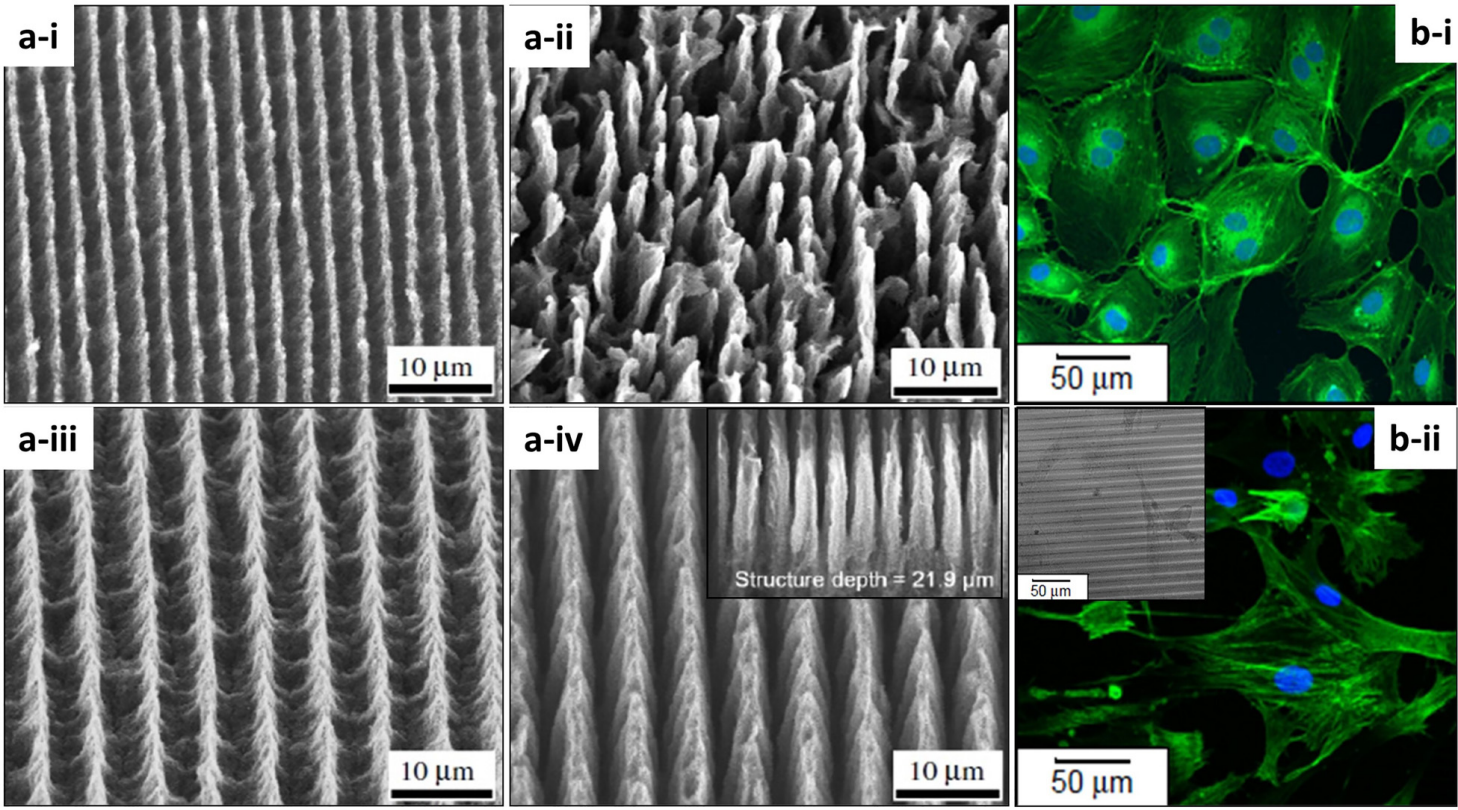
(a) SEM of one-dimensional vertical CNTs arrays produced (326 mJ cm^−2^) by interference pattern irradiation with (a-i) 2.86 µm spatial period and 1 pulse, (a-ii) 2.86 µm, *N* = 15, (a-iii) 5.73 µm, *N* = 1 and, (a-iv) 5.73 µm, *N* = 20 where it is showing the cross section of the linear arrays. (b) Confocal microscopy images with fluorescent labeling of smooth muscle cells cultured on DLC layers: (b-i) non-patterned slice and (b-ii) patterned slides with lamellipodia (elongation) response and SEM image of linear migration channels (upper left). Figures (a) and (b) adapted from ref. [186, 187] (© 2009 IOP Publishing).

In another work [186], periodic arrays on silicon films coated with vertical aligned multi-walled carbon nanotubes were fabricated using a ns-pulsed UV (355 nm) laser. The researchers stated that it was possible to obtain high aspect ratio structures by controlling the number of successive laser pulses (10–20). In addition, the repetitive overlap of laser pulses on the sample surface induced pattern distortion, leading to the formation of cone-shaped arrays. The SEM images of Figure 21(a) show the achieved structures for different spatial periods and number of pulses. The number of laser pulses required to achieve this distortion was proportional to the spatial period of the pattern. In this case, Raman spectroscopy analysis confirmed that the morphology of the carbon nanotubes was preserved after the laser processing structure. Marczak et al. [187] documented how a similar system of diamond-like carbon (DLC) film patterned by UV (266 nm) DLIP can potentially be used as scaffolds for targeted cell growth. Experiments were performed on hard, biocompatible 500 nm thick DLC substrates supported on an inert polymer and preliminarily tested on smooth muscle cell deposits. The fluorescence microscopy image in Figure 21(b-i) exhibits cell growth and migration in every direction on the unstructured surface, while the image in Figure 21(b-ii) hints at cell migration along directions parallel to the patterned grooves, giving rise to mature and enlarged cells.

Finally, it is worth mentioning a very recent work by Jurkevičiūtė et al. [188] where fs-DLIP was used for the fabrication of sub-micrometer grooves in DLC nanocomposites thin films doped with silver nanoparticles. By adjusting the fluence, the researchers controlled the line width of the grooves in the range from 150 to 420 nm, for a fixed spatial period of 564 nm. Furthermore, it was demonstrated that the resulting size distributions of silver nanoparticles in the composite (DLC:Ag) can be controlled by selecting the appropriate laser parameters during the DLIP process. For example, the DLC film with an Ag content of 14.1% and a bimodal mean effective nanoparticle size distribution of 17 and 46 nm (DLC:Ag-14) (11 mJ cm^−2^ laser fluence, 64,000 pulses) was transformed into unimodal with a mean diameter 14 nm by increasing the fluence (17 mJ cm^−2^, 64,000 pulses). Furthermore, the combined effect of the DLC:Ag material on the structure parameters was studied and it was concluded that the patterning of nanocomposite thin films requires a cumulative fluence 4 to 24 times higher to achieve structuring, with respect to their separate components (DLC and Ag). This is due to differences in the absorption and enhancement of the local electric field governed by the effects of localized surface plasmon resonances in the case of DLC:Ag.

## 5 Conclusions and perspectives

Over the last 23 years, the development of DLIP has made remarkable advancements towards establishing as a reliable industrial method for functionalizing surfaces. Apart from the huge work conducted on metallic samples, other materials, like polymers, ceramics, composites or diamond-like coatings, have been processed with this technique to achieve new or better surface properties. In this review, the physical and chemical processes that lead to melting and/or ablation on different non-metallic materials, such as ceramics or polymers, upon DLIP treatment with different laser sources have been briefly described. From this it follows that the intrinsic mechanical, optical and thermal properties of these materials as well as the characteristics of the used laser source need to be carefully considered for optimum DLIP processing.

From the cumulated experimental work on this field, it can be envisaged that these novel enhanced materials will become relevant for many innovative and emerging application areas, such as ceramic medical implants with optimized osseo-integration and antibacterial properties, polymeric electrodes for flexible optoelectronic devices with improved electrical and optical performance or in electroactive scaffolds for neural tissue engineering.
